# Geodynamically corrected Pliocene shoreline elevations in Australia consistent with midrange projections of Antarctic ice loss

**DOI:** 10.1126/sciadv.adg3035

**Published:** 2023-11-17

**Authors:** Fred D. Richards, Sophie L. Coulson, Mark J. Hoggard, Jacqueline Austermann, Blake Dyer, Jerry X. Mitrovica

**Affiliations:** ^1^ Department of Earth Science and Engineering, Imperial College London, London, UK.; ^2^ Fluid Dynamics and Solid Mechanics Group, Los Alamos National Laboratory, Los Alamos, NM, USA.; ^3^ Department of Earth Sciences, University of New Hampshire, Durham, NH, USA.; ^4^ Research School of Earth Sciences, Australian National University, Canberra, ACT, Australia.; ^5^ Lamont-Doherty Earth Observatory, Columbia University, Palisades, NY, USA.; ^6^ School of Earth and Ocean Sciences, University of Victoria, Victoria, BC, Canada.; ^7^ Department of Earth and Planetary Sciences, Harvard University, Cambridge, MA, USA.

## Abstract

The Mid-Pliocene represents the most recent interval in Earth history with climatic conditions similar to those expected in the coming decades. Mid-Pliocene sea level estimates therefore provide important constraints on projections of future ice sheet behavior and sea level change but differ by tens of meters due to local distortion of paleoshorelines caused by mantle dynamics. We combine an Australian sea level marker compilation with geodynamic simulations and probabilistic inversions to quantify and remove these post-Pliocene vertical motions at continental scale. Dynamic topography accounts for most of the observed sea level marker deflection, and correcting for this effect and glacial isostatic adjustment yields a Mid-Pliocene global mean sea level of +16.0 (+10.4 to +21.5) m (50th/16th to 84th percentiles). Recalibration of recent high-end sea level projections using this revised estimate implies a more stable Antarctic Ice Sheet under future warming scenarios, consistent with midrange forecasts of sea level rise that do not incorporate a marine ice cliff instability.

## Teaser

Mid-Pliocene shorelines, corrected for geodynamic vertical deflections, suggest high-end sea level projections are less probable.

## INTRODUCTION

Robust forecasts of future sea level change are dependent on our ability to accurately model the response of ice sheets to climate change. As atmospheric temperatures and CO_2_ concentrations continue to surpass those previously observed during human history, we must increasingly turn to the geological record of past warm periods to gain insights into ice sheet sensitivity (*1*). The Mid-Pliocene Warm Period (MPWP), approximately 3.3 to 3.0 million years (Ma) ago, is of particular interest because global mean temperature was 1.9 to 3.6°C above preindustrial levels and atmospheric CO_2_ concentrations were ∼400 parts per million, conditions comparable to those expected to prevail in the near future under many emissions scenarios(*2*–*5*). Although the rate of temperature and CO_2_ increase at the onset of the MPWP was likely lower than ongoing rises, estimates of global mean sea level (GMSL) during this period represent an important constraint on future ice sheet stability in the face of sustained warming (*1*, *6*).

An important problem with such an approach is that MPWP GMSL estimates exhibit significant variability between different studies. For example, ice sheet modeling indicates that GMSL was 4 to 13 m above present day (*7*, *8*), but values of up to +26 m can be obtained if the poorly understood ice sheet processes of meltwater-driven fracturing and ice cliff collapse [collectively known as the marine ice cliff instability (MICI)] are included (*1*). Alternatively, attempts to constrain paleo-ice volumes using temperature-corrected oxygen isotope records suffer from very large uncertainties (*9*), yielding MPWP GMSL estimates of +6 to +58 m (*10*–*12*).

The large uncertainties associated with these indirect constraints has led to renewed focus on the use of paleoshoreline elevations and other geological markers of former sea level to more directly constrain MPWP GMSL (*13*–*15*). Although these geomorphic estimates have, in many cases, been corrected for local uplift and subsidence, they span a range of +6 to +35 m (Table 1), indicating substantial and spatially variable vertical displacements of these features since their formation (*15*–*19*). These displacements have been variably attributed to sediment redistribution, tectonic activity associated with earthquakes and faulting, glacial isostatic adjustment (GIA; i.e., sea level variations caused by ice and ocean mass changes), and/or dynamic topography (i.e., vertical surface motions driven by mantle convection). Nevertheless, because previous studies have been limited to single sites or geomorphic features, the relative contribution of these different processes remains difficult to evaluate. Improving estimates of GMSL during the MPWP therefore requires the selection of a study area containing multiple field sites with reliably dated sea level markers where geodynamic and sedimentary processes can be either quantified with reasonable accuracy or assumed to have negligible impact. With these considerations in mind, we focus herein on Australian sea level records.

**Table 1.** Preexisting geomorphic proxy-derived estimates of MPWP GMSL. Initials represent ISO3166 country codes.

Location	Estimated GMSL range	Deposit type	Reference	Uplift/subsidence correction
Orangeberg Scarp (US)	15–35 m	Base of wave-cut scarp	(*23*, *87*)	+0–50 m uplift
Mallorca (ES)	17 (7–20) m*	Speleothem overgrowth	(*15*)	∼–1 m GIA and 1–15 m uplift
De Hoop Plain (ZA)	22–31 m	Base of wave cut scarp	(*13*, *19*)	No correction
Enewetak Atoll (MH)	20–25 m	Buried coral reef horizon	(*16*)	112–115 m subsidence
Wanganui Basin (NZ)	11 (6–17) m*	Backstripped sediments	(*18*)	No correction
Multiple locations†	22 (17–27) m*	Backstripped sediments	(*88*)	No correction

*50th (16th to 84th percentile).

†Virginia (US)/Wanganui (NZ)/Enewetak (MH).

## RESULTS AND DISCUSSION

### The Australian record of Pliocene sea level

In many respects, Australia represents an ideal setting for estimating GMSL from geological markers. The continent is surrounded by passive margins and is relatively remote from major plate boundaries (except in the far north where it encroaches within ∼1000 km of the Java and New Britain trenches). The most recent phase of continental rifting occurred between south Australia and Antarctica and had largely progressed to full seafloor spreading by Late Cretaceous times (*20*). Internal deformation, as judged from the modern distribution of seismicity, Neogene fault scarps, and borehole-breakout data, indicates only modest strain rates (*21*). Thus, tectonic deformation throughout the majority of the continent is minimal. Furthermore, away from the South Eastern Highlands and Flinders Ranges, Australia’s topography is dominated by low elevation and low relief, resulting in slow rates of erosion and sediment redistribution in comparison to other continents (*22*, *23*). Australia’s location in the far field of the former Laurentide and Fennoscandian ice sheets, in addition to Greenland, West Antarctica, and most marine-based sectors of East Antarctica, means that the GIA-induced change in sea level from Pliocene to present day is dominated by a signal proportional to any difference in total ice volumes across this period and a suite of more minor effects associated with remnant adjustment associated with the last glacial cycle (*4*). The latter includes ocean syphoning, the flux of water toward and away from peripheral bulges surrounding locations of ancient ice cover as these bulges subside and uplift across glacial cycles, and continental levering, the shoreline-perpendicular tilting of the crust and mantle driven by ocean loading and unloading (*24*). Of these effects, only levering introduces substantial geographic variability in sea level change across Australia, although this variability is limited to less than ∼5 m across coastal sites (see the “Calculating relative sea level change caused by GIA” section, section S4, and fig. S8) (*4*, *13*, *18*).

**Fig. 1.**
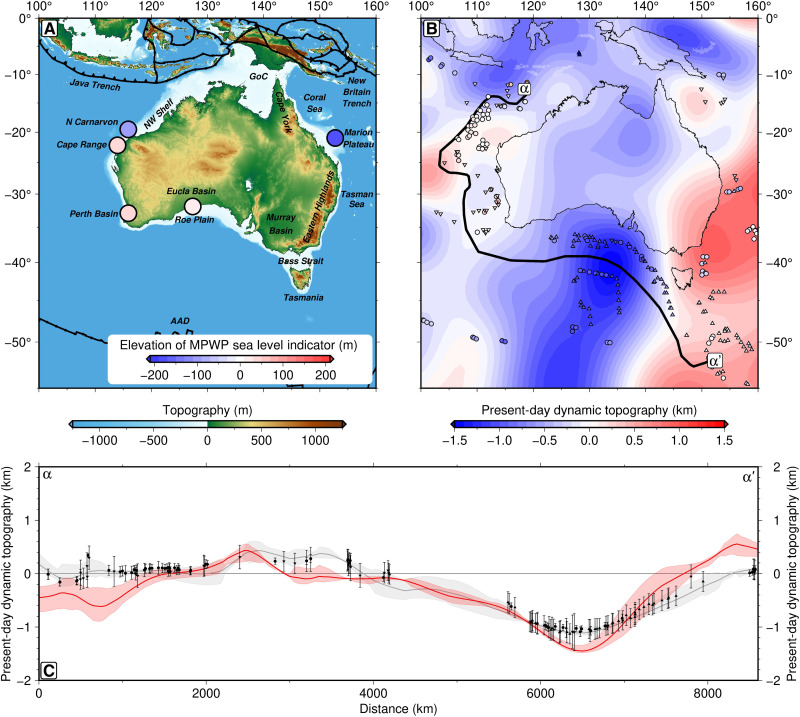
Australian Pliocene sea level markers and dynamic topography at the present day. (**A**) Location map of study region. Circles, markers colored by mean elevation of the paleo–sea level indicator (see Table 2); GoC, Gulf of Carpentaria; N Carnarvon, North Carnarvon Basin; AAD, Australian-Antarctic Discordance; NW Shelf, Northwest Shelf. (**B**) Predicted present-day dynamic topography from instantaneous mantle flow calculation for density structure derived from LLNL-G3D-JPS tomographic model (*37*) and the F10V2 mantle viscosity profile (*36*), optimized to fit global constraints on dynamic topography, geoid undulations, and core-mantle boundary (CMB) excess ellipticity (*34*). Colored circles/triangles, spot measurements of oceanic residual depth (a common proxy for observed dynamic topography) (*86*); thick black line, location of transect shown in (C). Predicted dynamic topography field is expanded up to spherical harmonic degree, *l*_max_ = 30. (**C**) Predicted versus observed present-day dynamic topography along northwest-to-southeast transect. Red line/band, prediction with uncertainties; circles/triangles with error bars, spot measurements of residual depth and uncertainties (*86*); gray line/band, spherical harmonic fit to spot measurements (*l*_max_ = 30). Uncertainty bands represent range within 500-km-wide swath perpendicular to transect.

Despite these factors, Late Pliocene geomorphic indicators record local sea levels that vary by approximately ±100 m around the continent (Fig. 1A and Table 2). These constraints fall into two broad categories. Onshore, MPWP paleoshoreline indicators are found in the Perth Basin [beach deposit at ∼40 m above sea level (m.a.s.l.)] (*25*, *26*), Cape Range (marine terrace at 15 to 40 m.a.s.l.) (*27*), and the Roe Plain (marine terrace at 15 to 30 m.a.s.l.) (*13*). The latter two have been dated to ∼2.7±0.3 Ma ago and ∼3.1±0.4 Ma ago, respectively, on the basis of strontium isotope analysis of bivalve shells, while the former is interpreted to be of Late Pliocene age (2.6 to 3.6 Ma ago) on the basis of biostratigraphic correlations. Offshore, relative sea level constraints include backstripped well data that record approximately −95 m of Pliocene-to-recent water-loaded elevation change in the North Carnarvon Basin (*28*) and −180 m on the Marion Plateau (see the “Compilation of relative sea level constraints” section for further details on each observation) (*29*). Given the relative tectonic quiescence, slow rates of sediment redistribution, and minor GIA impacts, this raises the question: Is dynamic topography responsible for this observed variability in Australian MPWP local sea level estimates? If so, can we accurately account for this dynamic topographic deformation? And what GMSL estimate do we obtain if we make a correction for both dynamic topography and GIA?

**Table 2.** MPWP sea level localities. Pl., plateau; Lat., latitude; Lon., longitude; Elev., elevation, PWD, paleo-water depth; Ref., Reference. Note that, for offshore markers, elevation uncertainty reflects compaction parameter uncertainties in backstripping procedure, while paleo-water depth represents change between MPWP and present instead of absolute paleo-water depth at time of deposition. This definition is equivalent in terms of corrected elevation, *e_c_* = *e* − *w_d_*, to that used onshore since, within error, water depth has not changed since the MPWP in these locations (i.e., if present-day water depth were included in *e* and paleo-water depth in *w_d_*, then these terms would cancel out when evaluating *e_c_*).

Locality	Lon.	Lat.	Elev. (m)	Elev. 1σ (m)	PWD (m)	PWD 1σ (m)	Age (Ma ago)	Age 1σ (Ma ago)	Ref.
Cape Range	113.98	−22.18	31	8	1	1	2.69	0.29	(*27*)
Perth Basin	115.91	−32.93	41	1	1	1	3.10	0.50	(*26*)
Roe Plain	127.36	−31.90	24	6	2	2	3.05	0.35	(*13*)
Marion Pl.	152.73	−20.97	−179	2	0	200	3.30	0.45	(*29*)
N Carnarvon	115.89	−19.52	−96	13	0	90	3.05	0.30	(*28*)

### Modeling Pliocene-to-recent mantle flow

Invoking an important role for dynamic topography in controlling Neogene vertical motions across Australia is not without precedent. Geological observations, including the uplift and subsidence of paleoshorelines in the Eucla and Murray basins, the width of continental shelves, stratigraphic geometries offshore, rapid subsidence of carbonate reefs on the Northwest Shelf, and volcanism and uplift of the Eastern Highlands as recorded by the fluvial geomorphological record have all previously been attributed to the spatiotemporal evolution of mantle flow beneath the continent (*28*–*33*). Nevertheless, before we can simulate the spatiotemporal evolution of Australian dynamic topography, we must first obtain models of the present-day mantle structure that are consistent with available geodynamic, seismic, and geodetic constraints.

We therefore adopt the approach of Richards *et al.* (*34*) to invert for mantle density models that simultaneously satisfy present-day estimates of dynamic topography, geoid height anomalies, core-mantle boundary (CMB) excess ellipticity, Stoneley modes, and semidiurnal body tides (see section S1 for details). These models include high-resolution upper mantle structure from surface wave tomography, account for anelastic effects and limited seismic resolution in the mid-mantle, and incorporate dense basal layers within the large low-velocity provinces (LLVPs). By varying the thickness and composition of the basal layer and predicting associated dynamic topography, geoid undulations, and CMB topography using instantaneous flow calculations, we obtain best-fitting density structures for 15 different combinations of radial viscosity profile [S10 (*35*), F10V1 (*36*), F10V2 (*36*), *V_S_* tomographic model LLNL-G3D-JPS (*37*), S40RTS (*38*), SAVANI (*39*), SEMUCB-WM1 (*40*), and TX2011 (*41*); table S1]. While the resulting geodynamic predictions provide good fit to observational constraints at a global scale, agreement between predicted dynamic topography and oceanic residual depth measurements varies regionally. Critically, this agreement is particularly strong around the margins of Australia (*r*= 0.76 to 0.85 for all models; Fig. 1, B and C; fig. S1; and table S1). This result confirms that our present-day mantle density models are relatively accurate beneath this region, thereby enabling us to hindcast mantle flow and associated changes in dynamic topography with some confidence.

To reconstruct the spatiotemporal evolution of Australian dynamic topography, we incorporate our suite of mantle density and viscosity models into numerical simulations of convection using the ASPECT software package (*42*, *43*). To more fully explore uncertainties in our reconstructions, rather than using only the 15 optimized mantle density models, we generate a 270-model ensemble based on the same five seismic tomographic and three radial viscosity inputs, but with two different LLVP dense-layer vertical extents, five dense-layer chemical density contrasts, and two plate motion histories (see the “Numerical modeling of mantle convection” section, section S2, and fig. S2). Although Australia does not directly overlie either the Pacific or African LLVP, their buoyancy does affect the long-wavelength pattern of mantle flow and spatiotemporal evolution of the geoid, both of which affect predictions of relative sea level change (*44*). We therefore also test a range of possible LLVP density structures. In all cases, free-slip boundary conditions are applied at the surface and CMB, with plate reconstructions used to rotate output fields such that dynamic topography change is calculated in a Lagrangian reference frame (see the “Calculating relative sea level change caused by dynamic topography” section). A prescribed plate-slip surface boundary condition was also tested but ultimately rejected, as the amplitudes and spatial patterns of predicted dynamic topography were inconsistent with observational constraints. This result suggests that imposing plate motions, rather than allowing them to naturally emerge from the simulated flow field, may introduce artifacts that degrade fit to observations (*45*). Such an outcome is perhaps to be expected since the relatively simple rheology used in our models cannot fully capture complex plate boundary interactions that influence the global plate motion circuit.

**Fig. 2.**
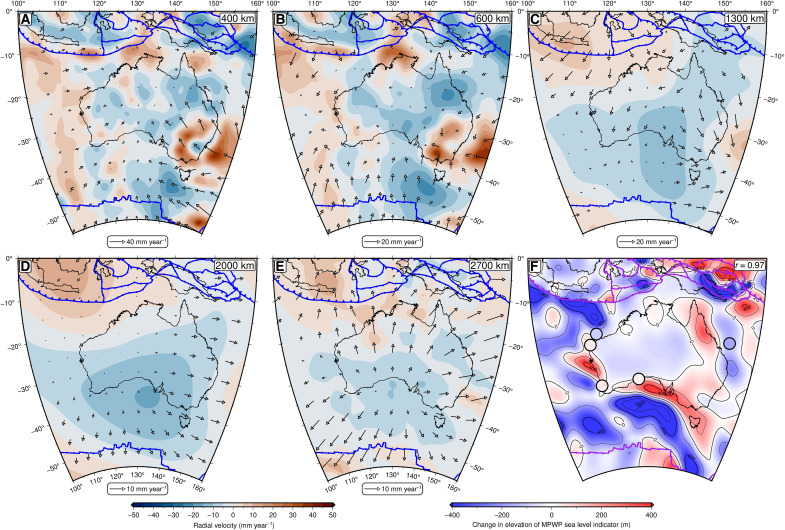
Predicted pattern of present-day mantle flow beneath Australia and associated post-MPWP dynamic topography change. (**A**) Radial component of mantle velocity at a depth of 300 km given by red-blue color scale; arrows, tangential component; blue lines, plate boundaries. (**B** to **E**) Same as (A), except at depths of 600, 1300, 2000, and 2700 km, respectively. (**F**) Predicted change in elevation of MPWP sea level markers due to dynamic topography evolution since 3 Ma ago. Circles, Mid-Pliocene median uncorrected GMSL estimates (i.e., present-day elevation + paleo-water depth; Table 2); purple lines, plate boundaries; *r*, correlation coefficient between predicted dynamic topography-induced elevation change and observed Mid-Pliocene median uncorrected GMSL estimates. Convection simulation based on LLNL-G3D-JPS tomographic model (*37*) and F10V2 viscosity profile (*36*).

Despite our relatively wide exploration of the parameter space, we nevertheless reconstruct consistent mantle flow patterns beneath Australia [representative examples are shown in Fig. 2, A to E, and figs. S3 and S4]. In all cases, the long-wavelength pattern is dominated by cold anomalies sinking beneath a region stretching from the Australian-Antarctic Discordance in the southwest to the Coral Sea in the northeast, with deep mantle return flow northeastward toward the Pacific LLVP. Hot upwellings rooted in both the lower mantle and mid-mantle are predicted beneath Cape Range, Cape York, Tasmania, and the Eucla Basin. High-viscosity Australian lithosphere travels rapidly northeastward over these flow structures, leading to strong shear-driven flow in the underlying asthenosphere (*46*). This motion leads to rapid changes in dynamic topography within the reference frame of the Australian plate (∼100 m Ma^−1^), with substantial increases in dynamic topography predicted across Cape York and from Cape Range counterclockwise around the coast into the Bass Strait (Fig. 2F). Predicted amplitudes and spatial patterns vary moderately as a function of model input, but the distribution of uplift and subsidence is remarkably similar (section S2 and figs. S5 to S7). This consistency is encouraging, as it indicates that comparable mantle flow histories are obtained beneath Australia for a range of tomographic models, suggesting that the resulting dynamic topography predictions are relatively reliable. Most of our simulated relative elevation changes also show good agreement with paleo–sea level observations (>50% yield a Pearson’s correlation coefficient, *r*, between 0.73 and 0.97), further strengthening our confidence that these dynamic topography simulations can be used to correct postdepositional warping of paleoshorelines on a continental scale. Last, although sea level observations are only available at five sites, the agreement of our predictions with indirect constraints on uplift and subsidence from seismic stratigraphy, river profile analysis, speleothem records, and the location of Neogene magmatism across Australia provides additional verification of our dynamic topography reconstructions (*29*–*33*, *47*). An important corollary of this evidence for rapid (∼0.1 km Ma^−1^) vertical motion is that it is essential to consider the impact of evolving dynamic topography on marker elevations in studies of former sea level (*13*, *48*–*50*).

### Reevaluating MPWP sea level

There are several important sources of uncertainty to consider when reconstructing GMSL using a suite of relative sea level markers. First, the age of the marker, the paleo-water depth in which it formed (i.e., its indicative range), and its present-day elevation are known to a limited degree of precision. Second, as previously discussed, Pliocene-to-recent plate motion history, mantle density, and mantle viscosity are imperfectly constrained, feeding into appreciable uncertainty in corrections for dynamic topography and GIA. In regard to the latter, variations in elastic lithospheric thickness and upper mantle viscosity have the largest impact on the magnitude of predicted sea level change due to their influence on continental levering (fig. S8). Last, benthic oxygen isotope constraints, backstripped sedimentary records, and coupled ocean–atmosphere–ice sheet model simulations suggest that substantial glacioeustatic sea level variations occurred during the MPWP (*18*, *51*, *52*). The exact amplitude and timing of these GMSL oscillations are, however, poorly constrained. Consequently, we have chosen to pose the determination of MPWP GMSL as a Gaussian process-based Bayesian inference problem, allowing these different sources of uncertainty to be robustly propagated into our final value. Within this framework, GMSL at time, *t*, is estimated from each sea level marker at longitude, ϕ, and latitude, θ, according to

$${\mathrm{GMSL}}_{\mathrm{obs}}(t)=e(\phi ,\theta )+w_{d}(\phi ,\theta )-C_{\mathrm{GIA}}(\phi ,\theta ,I,\eta ,t)-C_{\mathrm{DT}}(\phi ,\theta ,\rho ,\eta ,v,t)$$ (1)

where *e* is the present-day elevation, *w_d_* is the paleo-water depth, *C*_GIA_ is the correction for GIA (see the “Calculating relative sea level change caused by GIA” section for details of prediction), *C*_DT_ is the correction for dynamic topography (see the “Calculating relative sea level change caused by dynamic topography” section), *I* is ice history, η is mantle viscosity, ρ is mantle density, and *v* is plate motion history. A Gaussian process composed of a radial basis function (RBF) and a white noise kernel is then used to interpolate in time between these corrected sea level observations, providing a GMSL estimate that varies through time (the “Bayesian inference of Mid-Pliocene GMSL” section) (*53*). Given the large uncertainties in the magnitude and pacing of MPWP glacioeustatic cycles, instead of fixing parameters controlling the amplitude and wavelength characteristics of the time-dependent Gaussian process a priori, their most probable values are inferred directly from the input data. Posterior distributions for the different components of Eq. 1 are then sampled using a sequential Monte Carlo (SMC) algorithm.

Determining the uncertainty associated with the elevation, age, and paleo-water depth of each sea level marker is relatively straightforward and can be obtained from the associated field observations and laboratory analyses (see the “Compilation of relative sea level constraints” section). However, doing the same for the GIA correction and, in the case of Australia, the dynamic topography correction, requires knowledge of likely values of *C*_GIA_ and *C*_DT_ when *I*, *v*, ρ, and η are intermediate to the cases that we have already simulated. To avoid the computational expense of running thousands of additional simulations, we instead rapidly calculate their values using emulators (i.e., computationally efficient approximations of the full numerical simulations). These emulators are constructed by training two separate neural networks on synthetic data derived from the existing GIA and dynamic topography simulations (see the “Constructing neural network emulators” section). In both cases, 10% of the input data are excluded from the training process, allowing us to assess the ability of the networks to accurately predict GIA and dynamic topography fields for previously unseen input parameters. Once trained, these feed-forward networks can be incorporated into the SMC algorithm, allowing uncertainty associated with geodynamic processes to be characterized. This approach enables the model outputs that better explain observed spatial variability in relative sea level change to be effectively upweighted in a statistically robust manner since they will naturally be sampled more frequently due to their superior likelihood.

**Fig. 3.**
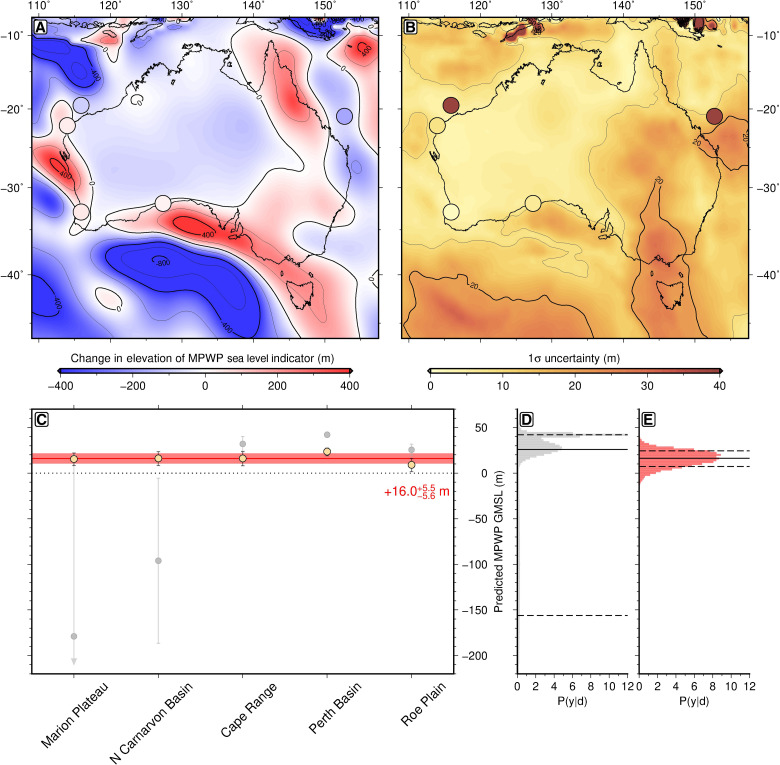
Correcting MPWP relative sea level markers for mantle dynamics. (**A**) Median-predicted change in MPWP sea level marker elevation. Background color, median of 3 Ma ago–to–present combined dynamic topography (DT) and GIA posterior probability distribution. Circles, Mid-Pliocene median uncorrected GMSL estimates (i.e., present-day elevation + paleo-water depth; Table 2). (**B**) Uncertainty on predicted elevation change. Background color, 1σ uncertainty of 3 Ma ago-to-present combined DT and GIA posterior distribution. Circles, 1σ uncertainty of Mid-Pliocene uncorrected GMSL estimates. (**C**) DT- and GIA-corrected Mid-Pliocene GMSL along transect counterclockwise from Cape Range. Yellow circles/error bars, 50th/16th to 84th percentiles of DT- and GIA-corrected posterior distribution. Gray circles/error bars, same for uncorrected prior distribution. (**D**) Histogram of uncorrected Mid-Pliocene GMSL prior distribution. Solid/dashed lines, 50th/16th to 84th GMSL percentiles. (**E**) Same for DT- and GIA-corrected Mid-Pliocene GMSL posterior distribution.

**Fig. 4.**
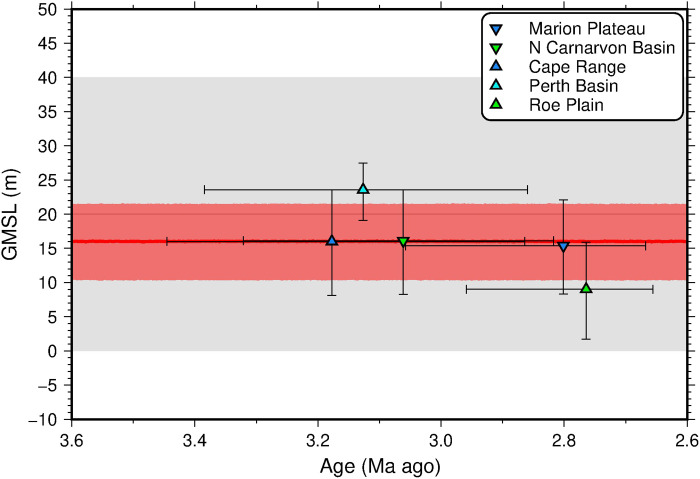
Probability distributions for MPWP GMSL. Gray line/band, 50th/16th to 84th percentiles of prior probability distribution for MPWP GMSL Gaussian process; red line/band, 50th/16th to 84th percentiles of posterior distribution; colored symbols, posterior age and GMSL estimates and errors for individual marker sites (see legend).

The Bayesian inversion scheme yields a revised MPWP GMSL of ${+16.0}_{-5.6}^{+5.5}$ m (section S4; Figs. 3, C to E, and 4; and fig. S9 to S11). This range represents a 70% drop in uncertainty with respect to the prior MPWP GMSL estimate (∼20 ± 20 m), demonstrating that, despite substantial uncertainties on individual model parameters, our data compilation and model predictions provide sufficient information to significantly improve constraint on GMSL during the MPWP. This estimate is the first to successfully reconcile MPWP sea level marker elevations across multiple widely separated sites at continental scale. In addition, by adopting a probabilistic framework in which geodynamic model parameter uncertainties are propagated into the final inference, we can quantitatively assess which parameterizations of Earth’s internal structure and evolution are more likely to be accurate, while simultaneously placing more robust bounds on the plausible range of peak MPWP GMSL (section S4). Both features of the approach improve confidence in the GMSL estimate, but this result will require corroboration in other regions.

Individual GMSL histories sampled from the fitted Gaussian process produce peak-to-trough GMSL variations of $4.7_{-3.3}^{+4.7}$ m (50th and 16th to 84th percentiles) but predict minimal GMSL change at periods greater than ∼40 thousand years (ka). The absence of any signal in this period range can be partly attributed to the prior distribution used for the timescale parameter of the Gaussian process. Nevertheless, we find that increasing the prior mean by an order of magnitude to 20 ka has no impact on the inferred posterior GMSL distribution. This result suggests that, while it is possible that our Mid-Pliocene paleoshoreline dataset records more than one sea level highstand, such long-period GMSL variability cannot be reliably retrieved from our analysis. This factor is likely due to the small size of our input dataset and its relatively large age uncertainties. Consequently, the 16th, 50th, and 84th percentiles of the inferred GMSL history remain relatively consistent throughout the MPWP, and most of the short-period variability is cancelled out as a result of changes in the phase of predicted sea level cycles from one sampled GMSL function to the next (Fig. 4).

By correcting for +1.2 ± 0.6 m of thermosteric sea level change [assuming a contribution of +0.2 to 0.6 m °C^−1^ (*54*)], our revised MPWP GMSL range (+10.4 to 21.5 m) can be converted into an estimate of ice volume loss relative to the modern state, expressed as meters of GMSL equivalent (GMSLE). The resulting ∼15-m median value suggests a considerable loss of ice from Greenland and West Antarctica, with the possibility of minor mass loss from East Antarctica. By contrast, the ∼20-m upper bound would require considerable additional loss of marine-based ice in East Antarctica (*55*). The full +9.2- to 20.3-m GMSLE range is consistent with other recent estimates of MPWP ice loss, including +5.6- to 19.2-m GMSLE from speleothem overgrowths in the western Mediterranean (*15*), +5.0- to 15.5-m GMSLE from backstripped sea level records in New Zealand (corrected assuming the same +1.2-m thermosteric contribution) (*18*), and +4- to 13-m GMSLE from ice sheet models that exclude MICI processes (*7*, *8*). It is, however, toward the lower end of most estimates that are based on analysis of oxygen isotopes in benthic foraminifera [e.g., +16.3- to 38.1-m GMSLE (*10*), +11.2- to 33.3-m GMSLE (*12*), and +11.1- to 31.2-m GMSLE (*11*); all corrected with a +1.2-m thermosteric contribution].

### Implications for predictions of future sea level rise

Our MPWP GMSL estimate of ${+16.0}_{-5.6}^{+5.5}$ m is consistent with partial collapse of polar ice sheets and has important ramifications for recent studies predicting future sea level change. For example, the recent ice sheet model–based sea level projections of DeConto *et al.* (*1*) are calibrated using an assumed MPWP sea level contribution of 11 to 21 m from Antarctica alone. That study found that it is necessary to include both a marine ice sheet instability and strong MICI processes to obtain such substantial ice loss under MPWP climatic conditions. If Paris Agreement targets are exceeded, then these authors showed that applying these same parameterizations of ice sheet behavior to future melting scenarios leads to potentially rapid and irreversible sea level rise. Although we note that certain models from other studies have generated similarly large MPWP Antarctic contributions without requiring the operation of MICI (*56*), the high-end DeConto *et al.* (*1*) projections underpin the “worst case” sea level rise scenarios currently used by decision-makers in the development of coastal flood risk management plans.

**Fig. 5.**
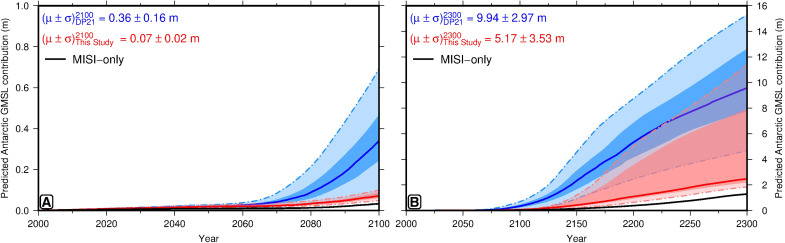
Impact of revised MPWP GMSL estimate on future sea level predictions. (**A**) 2000–2100 Antarctic GMSL contributions under RCP8.5 (high-emissions scenario) based on simulations of DeConto *et al.* (*1*). Blue, projections consistent with their original +16 ± 5 m MPWP Antarctic contribution; red, same for our revised value of ${+9.8}_{-5.7}^{+5.6}$ m; black, projections for ice sheet models that exclude the MICI mechanism. Solid lines and dark/light shading, ensemble median and 50%/99% confidence intervals. (**B**) Same for 2000–2300 period.

Accounting for the inferred extent of the Mid-Pliocene Greenland ice sheet (thought to be equivalent to 5 ± 1-m GMSLE compared to present day) (*52*) and a 1.2 ± 0.6-m thermosteric increase in sea level, our revised estimate for the Antarctic Ice Sheet contribution is ${+9.8}_{-5.7}^{+5.6}$ m GMSLE (total uncertainty is calculated by propagating that of individual contributions under the assumption that they are mutually independent and uncorrelated). These values are broadly consistent with previous estimates of Mid-Pliocene Antarctic contributions to GMSL (*56*); however, there is no overlap with the upper half of the DeConto *et al.* 11- to 21-m MPWP range. Since their model outputs for the MPWP and future are publicly available, we are able to quantify the impact of our revised MPWP GMSL estimate on recent high-end sea level projections by repeating their calibration process using our upper bound of ≤+15.4 m (see the “Recalibration of sea level projections” section). We find that the range of simulations that are consistent with observational targets narrows markedly (only 15 of their models pass versus the original 109). Restricting ourselves to this reduced model ensemble leads to substantially slower and lower magnitude Antarctic contributions to future sea level rise (i.e., the most extreme melting scenarios are excluded). Projected end-of-century GMSL rise under the RCP8.5 emissions scenario decrease by ∼70% from ${+34}_{-14}^{+21}$ cm (50th/16th/84th percentiles) to ${+7}_{-1}^{+2}$ cm (Fig. 5A). By 2300, the original and revised RCP8.5 ensemble projections become more comparable, but the median estimate (i.e., 50th percentile) remains ∼70% smaller (approximately +2.5 m versus +9.6 m; Fig. 5B).

These updated end-of-century Antarctic sea level predictions overlap with recent ensemble projections of ice sheet models that do not incorporate the MICI mechanism [${+4}_{-5}^{+6}$ cm for RCP8.5 (50th/16th/84th percentiles)] (*57*). In addition, our +16-m median MPWP GMSL estimate, which implies an Antarctic sea level contribution of ∼10-m GMSLE, also agrees well with Mid-Pliocene ice sheet simulations that exclude MICI [e.g., 9.8 ± 2.1 m (*58*) and 7.8 ± 4.0 m(*56*)]. Although uncertainties in climate and ice sheet models mean that caution is warranted when interpreting associated sea level projections, these results suggest that it may not be necessary to invoke MICI processes to explain either MPWP ice volumes or to predict future Antarctic Ice Sheet contributions to sea level change. Therefore, although the West Antarctic Ice Sheet may still be susceptible to runaway disintegration on multicentennial time scales, our work indicates that recent high-end projections envisaging a >20-cm end-of-century Antarctic sea level contribution under RCP8.5 and SSP5-85 emissions scenarios are less probable (*1*). Instead, our results are consistent with the midrange (i.e., "likely") predictions of the Intergovernmental Panel on Climate Change (IPCC) Special Report on the Ocean and Cryosphere in a Changing Climate, which predict a ${+12}_{-9}^{+16}$ cm (50th/16th/84th percentiles) Antarctic contribution and a $84_{-23}^{+26}$ cm GMSL rise under these emissions trajectories (*59*). While our analysis is globally applicable, it is currently based on sea level marker sites in Australia. With further improvement in paleoshoreline datasets and models of present-day Earth structure, our MPWP GMSL estimate can be verified in other regions remote from major ice sheets. Given the demonstrated implications of this constraint for ice sheet dynamics in a warmer climate, we would suggest that this validation is an important goal for future research.

## MATERIALS AND METHODS

### Compilation of relative sea level constraints

Allrelative sea level constraints used in this study have been compiled from preexisting publications. The present-day elevations of Cape Range and Roe Plain MPWP sea level markers have been accurately measured using differential GPS (DGPS; ≤1-m uncertainty). In the case of the Roe Plain, the paleoshoreline elevation is defined as 24 ± 6 m based on the spread of DGPS-derived scarp toe-line elevations sampled at Madura Quarry, Elarbilla, Carlabeencabba, and Boolaboola (*13*). We restrict ourselves to using these DGPS-derived values since, unlike those inferred from digital elevation models, they have been groundtruthed via direct field survey and have significantly lower uncertainty. Similarly, at Cape Range, a paleoshoreline elevation of 31 ± 8 m is derived from local averages of the three highest DGPS-sampled marine-limiting features on the Milyering Terrace and contacts between the Tulki Limestone and Exmouth Sandstone units (*27*). In the Perth Basin, the Mid-Pliocene paleoshoreline is defined by the toe-line of the Whicher Scarp at an elevation of 41 ± 1 m, as inferred from Shuttle Radar Topography Mission data (30-m resolution) (*26*). These estimates encompass along-scarp variations in elevation; we make no attempt to fit these local undulations because they are shorter wavelengths than can be reliably resolved via our seismic tomography-based mantle flow models (<200 km) and may instead represent unmodeled processes, such as neotectonics and sediment loading, which we treat as geological noise in our inversion scheme.

Offshore sea level constraints are derived from backstripped well data. In each case, the local water-loaded elevation changes recorded by Mid-Pliocene horizons are obtained by correcting for postdepositional sediment loading and compaction using the approach outlined in Kominz *et al.* (*60*), assuming Airy isostatic compensation. Since active rifting ceased between ∼70 and 160 Ma before present at each well location, Mid-Pliocene–to–recent thermal subsidence is assumed to be negligible and no correction is made for this process. Data from the North Carnarvon Basin (−96 ± 91 m) are taken from Czarnota *et al.* (*28*), while the Marion Plateau constraint (−179 ± 200 m) is derived from DiCaprio *et al.* (*29*) These offshore values include uncertainties in both paleo-water depths and sediment compaction parameters.

### Numerical modeling of mantle convection

Our time-dependent mantle convection simulations use the finite-element software, ASPECT (Advanced Solver for Problems in Earth’s ConvecTion), which solves the coupled equations governing conservation of mass, momentum, and energy (*42*, *43*). Solving these equations for time-evolving changes in temperature, velocity, and pressure requires the specification of several boundary and initial conditions to produce a starting temperature, density, and viscosity structure, as well as parameterizations for the rheological properties that govern their subsequent evolution.

#### *Temperature structure*

In our simulations, the initial temperature field is determined using a hybrid approach. In the upper mantle, temperature anomalies above 400 km are derived from a modified version of the RHGW20 temperature and density model (*61*), which accounts for anelasticity at seismic frequencies and has been demonstrated to yield acceptable fits to present-day short-wavelength dynamic topography. Unlike RHGW20, which is based exclusively on the SL2013sv global surface wave tomographic model (*62*), the upper mantle model that we adopt here is augmented with regional high-resolution tomographic studies in North America [SL2013NA (*63*)], Africa [AF2019 (*64*)], and South America and the South Atlantic Ocean [SA2019 (*65*); see Hoggard *et al.* (*66*) and Richards *et al.* (*34*) for further details]. Although, incorporating these high-resolution regional models does not affect inferred mantle structure beneath Australia, associated improvements in global dynamic topography and geoid predictions enhance the accuracy of calculated relative sea level changes along the Australian margin. The lithosphere-asthenosphere boundary is delimited using the 1200°C isothermal surface, and we assume that temperature decreases linearly from this interface to the surface. Note that in the continental lithosphere, this thermal structure is adapted to produce neutral overall buoyancy (see the following section).

Below 300 km, temperatures are derived from thermodynamic modeling. Following Austermann *et al.* (*67*), we assume a pyrolytic background mantle composition and use Perple_X alongside the thermodynamic database of Stixrude and Lithgow-Bertelloni (*68*) to generate a lookup table of anharmonic shear-wave velocities and densities, varying temperature from 300 to 4500 K in 50 K increments and pressure from 0 to 140 GPa in 0.1-GPa increments. At each depth, temperature-dependent discontinuities in density and seismic velocity caused by phase transitions are smoothed by adopting the median temperature derivative across a ±500°C swath either side of the geotherm (*69*). Smoothed anharmonic velocities are then corrected for anelasticity using a *Q* profile determined using the approach of Matas and Bukowinski (*70*), as outlined in Richards *et al.* (*34*). Having smoothed and corrected the *V_S_* lookup table, velocities from five different seismic tomographic models—LLNL-G3D-JPS (*37*), S40RTS (*38*), SAVANI (*39*), SEMUCB-WM1 (*40*), and TX2011 (*41*)—are converted into temperature, with values adjusted by a constant offset to ensure that mean temperatures are consistent with the mantle geotherm (*69*). Note that, following Richards *et al.* (*34*) and Davies *et al.* (*71*), we high-pass filter the seismic velocity models within the 1000 to 2000 km depth range to correct for vertical smearing of long-wavelength structure and thereby obtain an acceptable fit to the observed long-wavelength geoid-to-topography ratio. This filtering is accomplished by multiplying the spherical harmonic coefficients, *c_lm_*, of the seismic velocity fields with a monotonic truncation function, *f*(*l*) that increases smoothly from 0 to 1 with spherical harmonic degree according to

$$f(l)=\left\{ \begin{array}{cc} -{\left(\frac{l-l_{\min}}{l_{\max}-l_{\min}}\right)}^{4}+2{\left(\frac{l-l_{\min}}{l_{\max}-l_{\min}}\right)}^{2} & \mathrm{for}\;l\leq l_{\max}\\ 1 & \mathrm{for}\;l>l_{\max} \end{array}\right.$$

where *l*_min_ = 1 is the minimum spherical harmonic degree in the truncation [at which *f*(*l*) = 0] and *l*_max_ = 8 is the maximum degree [at which *f*(*l*) = 1]. Between 300- and 400-km depth, temperatures derived from the two parameterizations are smoothly merged by taking their weighted average.

#### *Mapping temperature into density*

To self-consistently convert these initial temperature fields into density distributions within ASPECT, we construct a radially averaged thermal expansivity profile that is compatible with both our upper and lower mantle *V_S_*-to-density parameterizations (fig. S2). We also simplify our model calculations by assuming incompressible convection and therefore remove adiabatic increases in temperature and density with depth. Since heat flow measurements, xenolith geochemistry, seismic velocity, gravity, and topography observations suggest that compositional and thermal density contributions approximately balance each other within the continental lithosphere (*72*, *73*), we make these regions neutrally buoyant by resetting their temperature to the average of all external material at the relevant depth. Last, following Richards *et al.* (*34*), we investigate the potential impact of chemical heterogeneity in the lowermost mantle by defining the bottom 0 to 200 km of LLVP regions as a separate compositional field with an excess density ranging from 0 to 132 kg m^−3^ (0 to 4% of the 3330 kg m^−3^ reference density, ρ_0_).

Our mapping from temperature to density can therefore be expressed using

$$\rho (z,T,C)=\rho_{0}[1-\alpha (z)(T^{\prime }-T_{0})]+\text{Δ}\rho_{C}C$$ (2)

where Δρ*_C_* represents compositional excess density, *C* is the compositional field index (*C* = 1 inside the LLVP basal layer; *C* = 0 elsewhere). α(*z*) represents the radial thermal expansivity profile (fig. S2), *T*_0_ = 1600 K is the reference temperature, and *T*′ represents the temperature after subtraction of the adiabat [*T*′ = (*T* − *T_ad_*) + *T*_0_]. Note that in cases where either Δρ*_C_* or the basal layer thickness is equal to zero, *C* is set to zero throughout the model domain (i.e., these simulations are isochemical). In total, this approach generates 45 separate density models comprising different combinations of tomographically inferred initial temperature distribution, dense basal layer thickness, and compositional density anomaly.

#### *Viscosity structure*

Viscosity in each convection simulation is parameterized using three different radial profiles, η*_r_*(*z*), [S10 (*35*); F10V1 and F10V2 (*36*)], with lateral variations in viscosity incorporated using

$$\eta (z,T)=\eta_{0}(z)\varepsilon_{C}C\exp[-\varepsilon_{T}(z)(T-T_{0})]$$ (3)

where ε*_T_*(*z*) is the thermal viscosity exponent [ε*_T_*(*z*) = 0.01 for 0 km ≥ *z *≥ 670 km; ε*_T_*(*z*) = 0.005 for 670 km > *z *≥ 2891 km], η_0_(*z*) represents the prescribed radial viscosity profile, and ε*_C_* = 100 represents the compositional viscosity prefactor. The three radial viscosity profiles are selected on the basis of recent work showing that seismic, geodynamic, and geodetic observables can be simultaneously reconciled using these inputs and assuming a chemically distinct basal layer within the LLVPs (*34*). The upper mantle ε*_T_* value is chosen on the basis of the dual need to generate a realistically stable sublithospheric thermal boundary layer and to be consistent with experimental constraints on the activation energy of mantle rock. A value of 0.01, which is consistent with low-end experimental activation energy estimates (∼200 kJ mol^−1^), achieves both aims. A smaller lower mantle ε*_T_* is used because independent studies indicate that the diffusion creep activation energy of perovskite is approximately half that of olivine (*74*). In addition, we found that adopting an identical value to the upper mantle led to unrealistically low deep mantle viscosities and rapid upwelling of lower mantle structure, which degraded fit to both Mid-Pliocene and present-day observables. The ε*_C_* value (100), which applies to models in which the basal layers of LLVPs contain compositional anomalies (*C* = 1), is chosen on the basis that these regions likely contain smaller proportions of low-viscosity post-perovskite and larger volumes of high-viscosity silicic phases (e.g., stishovite and seifertite) compared to background mantle material (*34*). This inference is further supported by a recent study that demonstrated that geoid observations are better matched by model predictions when LLVP material is assigned a similar viscosity to its surroundings, indicating that thermal and compositional controls on viscosity may counterbalance one another in the lowermost mantle (*71*).

#### Numerical model parameterization

Equipped with these temperature, density, and viscosity inputs, we predict the time-dependent evolution of mantle circulation over the past 5 Ma using the backward advection method. This approach solves the governing equations in a forward sense, but with the sign of gravity reversed and thermal conductivity set to zero because thermal diffusion is numerically unstable when reversed in time. The resulting absence of a diffusive term in the energy equation does progressively reduce numerical solution accuracy with each time step; however, this scheme has been shown to yield valid results over ≤30-Ma simulation periods (*75*) and considerably reduces computational expense relative to other “retrodiction” methods, enabling a fuller exploration of density and viscosity uncertainties. Since ASPECT does not include self-gravitation, we impose the radially varying gravity profile from Glišović and Forte (*76*), while heat capacity is set to a constant value of 1250 J K^−1^ kg^−1^. Three surface boundary condition options were tested initially, including prescribed plate-slip, free-slip and no-slip. A free-slip condition was found to maximize agreement between predicted and observed present-day dynamic topography, as well as Pliocene-to-recent relative sea level change, so this assumption was applied in all models in our main ensemble. These simulations also assume a free-slip boundary condition at the CMB. In the upper 1000 km of the mantle, our numerical grid has ∼30-km radial resolution, increasing to ∼90 km below this depth, while lateral resolutions in the same depth ranges are ∼80 km and ∼210 km, respectively. This resolution is achieved using an initial global mesh refinement of 4 and an adaptive refinement of 1 applied only to mesh points shallower than 1000-km depth.

### Calculating relative sea level change caused by dynamic topography

UsingASPECT, we calculate dynamic topography, *h*, at each time step of our simulation from the predicted normal stress, σ*_rr_*, applied to the surface using

$$h=\frac{\sigma_{rr}}{(g\cdot n)\text{Δ}\rho }$$ (4)

where (**g** · **n**) is the component of gravitational acceleration normal to the upper boundary and Δρ is the density difference between outer grid cells and the overlying material, assumed to be air in the ASPECT calculations (note that water loading in oceanic regions is accounted for in postprocessing steps described below). To determine dynamic topography changes as a function of time at specific sites, it is important to account for plate motions over the intervening time span. We do so by applying two different plate motion reconstructions, one based on geological and GPS measurements [MORVEL(*77*)] and the other on magnetic anomalies [S12 (*78*)], to translate the dynamic topography field calculated for each time period into its present-day coordinates before subtracting the rotated paleo-dynamic topography field from its present-day equivalent. By calculating these outputs for each convection simulation and plate motion reconstruction, 270 separate dynamic topography histories are generated overall. To directly compare predicted dynamic topography changes to Mid-Pliocene relative sea level observations, we also account for changes in water loading caused by mantle dynamics. This correction adopts the framework described in Austermann and Mitrovica (*44*), which accounts for relative sea level change arising from the predicted evolution of dynamic topography and associated geoid undulations at each time step.

### Calculating relative sea level change caused by GIA

GIA-inducedchanges in relative sea level since the MPWP are calculated using the ice-age sea level theory and pseudo-spectral algorithm (truncated at spherical harmonic degree and order 256) of Kendall *et al.* (*79*), as implemented in Raymo *et al.* (*4*). The calculations require the Earth’s depth-varying rheological structure to be specified in addition to an MPWP-to-recent ice-loading history. We test two distinct ice sheet loading histories. The first assumes that West Antarctica and Greenland were completely deglaciated prior to 2.95 Ma ago, while the East Antarctic Ice Sheet had an equivalent volume to the present-day ice sheet. After this time, the West Antarctic and Greenland ice sheets rapidly grew to present-day thicknesses. The second, by contrast, assumes a Mid-Pliocene ice sheet configuration identical to the present day. After 2.95 Ma ago, both reconstructions assume that ice volume varies according to scaled δ^18^O from the LR04 benthic stack (*51*), with the corresponding geographic distributions based on ICE-5G model (*80*) time slices during periods with comparable δ^18^O values. From the LIG to present day, ice volume varies according to the ICE-5G reconstruction. These two ice sheet histories are paired with two different radial viscosity profiles to determine the spatially variable changes in relative sea level since the MPWP caused by GIA: VM2 (90-km elastic lithosphere, ∼5 × 10^20^ Pa·s upper mantle viscosity, and 2 × 10^21^ to 3 × 10^21^ Pa·s lower mantle viscosity) and LM (120-km elastic lithosphere, ∼5 × 10^20^ Pa·s, upper mantle viscosity, and 5 × 10^21^ Pa·s lower mantle viscosity).

Our GIA simulations based on these inputs incorporate time-varying shorelines owing to local flooding and regression, the growth and deglaciation of grounded marine-based ice sheets, the associated migration of water into or out of these marine settings, and the feedback between sea level and perturbations of Earth’s rotation vector (*79*, *81*). Note that we remove the GMSL produced by each reconstruction (either 0 or 14 m) because we aim to reconcile spatial variations in relative sea level markers while making no prior assumption about paleo-ice volume.

### Constructing neural network emulators

To generate reasonable dynamic topography predictions for combinations of density, viscosity, and plate motion inputs that are intermediate to those of our 270 numerical simulations, we train a neural network using synthetic data drawn from these simulations. Ten percent of this input data are held back from the training process, allowing us to later validate the performance of the network on data that it has not learned from. The remaining 90% is fed into a neural network with three fully connected dense layers containing 512 nodes with rectified linear unit activation functions. The final output layer has linear activation and produces a one-dimensional vector containing the dynamic topography prediction for a given input parameter set.

By comparing network predictions with target outputs for a known set of input parameters, backward propagation of errors is used to train the weights and biases in the network layers to improve performance. Input parameters for the model include indices for each tomographic model [0–1], indices for each viscosity profile [0–1], a plate motion index [0–1], LLVP dense layer thickness [0 to 200 km], chemical density difference [0 to 132 kg m^−3^], age [2 to 4 Ma ago], latitude [7.368° to 46.667°S], and longitude [108.98° to 157.5°E]. To improve learning efficiency, we first normalize these inputs to have zero mean and unit standard deviation. The network is subsequently optimized using an adaptive learning rate algorithm known as Adam (*82*). Training was halted after 500 epochs, where training and validation loss reached ∼1 m and showed no further improvement over a 20-epoch period. The set of learnt weights and biases associated with the minimum value of validation loss are then hard-coded into our Bayesian inverse modeling framework to rapidly simulate dynamic topography for any random sample of input parameters.

A similar approach is taken to emulate GIA predictions for models with viscosities and ice histories in between the four end-member combinations used in our full numerical simulations. However, since the synthetic training dataset available in this case is smaller, we found that it was necessary to modify our network structure to include 20% dropout layers between each of the three dense, fully connected layers. Training for this network is stopped after 100 epochs when validation loss ceased to improve beyond its minimum value of ∼0.5 m.

### Bayesian inference of Mid-Pliocene GMSL

We evaluate GMSL during the MPWP using a Bayesian Gaussian process regression framework that integrates our MPWP relative sea level constraints, and their associated age, elevation, and water depth uncertainties, with the trained weights and biases of our dynamic topography and GIA neural network emulators. The principal advantage of this approach is that it enables uncertainties associated with both observations and predictions of postdepositional geodynamic processes to be propagated into our assessment of GMSL in a statistically robust manner.

It is assumed that GMSL variation as a function of time, *f*(*t*), can be approximated by a Gaussian process comprising a RBF, and a mean function, μ(*t*), using the expression

$$f(t)∼GP[\mu_{GP}(t),k_{RBF}(t,t^{\prime })]$$ (5)

*k*_RBF_ is the RBF kernel, which takes two input points, *t* and *t*′, and calculates a similarity measure between the two in the form of a scalar according to

$$k_{\mathrm{RBF}}(t,t^{\prime })=\sigma_{\mathrm{GP}}^{2}\exp\left(-\frac{\left\|{\left(t-t^{\prime }\right)}^{2}\right\|}{2\lambda_{\mathrm{GP}}^{2}}\right)$$ (6)

where $\sigma_{\mathrm{GP}}^{2}$ is the variance of the function and λ_GP_ is the time scale. The GMSL observations, GMSL_obs_(*t*) from Eq. 1, are then assumed to represent the unknown function *f*(*t*) plus random noise, ε of the form

ε∼N(0,Σ) (7)

yielding the relationship between the Gaussian process and the observations

$${\mathrm{GMSL}}_{\mathrm{obs}}(t)=f(t)+\varepsilon$$ (8)

Instead of fixing their values, we set prior distributions for the Gaussian process parameters. The time scale prior (λ_GP_) is an inverse Gaussian distribution with mean μ = 2 ka and shape parameter λ = 5 ka, thereby encoding the assumption that any Mid-Pliocene sea level variability recorded in our constraints is on the typical interglacial time scale of a few thousand years. A normal distribution with μ = 0 m and σ = 1 m is used as a prior for the standard deviation (σ_GP_), thereby allowing for modest MPWP GMSL variability on interglacial time scales without presupposing its presence. The mean (μ_GP_) is assigned a Gaussian prior with μ = 20 m and σ = 20 m based on the range of preexisting estimates of MPWP sea level from previous studies (Table 1). The prior for the noise scaling parameter, Σ, is a half-normal distribution (i.e., positive values only) with σ = 2 m. Age, water-depth, and elevation uncertainties are assumed to be Gaussian, with means and standard deviations summarized in Table 2. Uniform priors were assumed for all emulator inputs, with an additional constraint in the case of the dynamic topography emulator that the sum of the five tomographic model indices and that of the three viscosity profile indices must both be equal to unity (i.e., total model contributions must sum to 100%).

Posterior GMSL distributions are calculated using an SMC algorithm implemented via the probabilistic programming package PyMC3 (*83*), with likelihood of a given parameter sample determined on the basis of cumulative misfit between the associated Gaussian process function and individual relative sea level observations corrected for water depth, GIA, and dynamic topography [i.e., GMSL_obs_(*t*)]. This methodology is chosen for its ability to fully sample the potentially multimodal probability distributions that we might expect for certain of the input parameters. To ensure sufficiently dense sampling, we compute six independent SMC chains, each with 2000 draws, and evaluate the resulting Gelman-Rubin and effective sample size statistics to confirm convergence of the algorithm. We further validated our approach via tests conducted on synthetic data generated from a prescribed GMSL function, randomly selected GIA and dynamic topography predictions, and the elevation, age, and water depth uncertainties of the relative sea level observations. In these tests, prescribed GMSL lies within the ±1σ region of the GMSL posterior derived from the synthetic observations, confirming the robustness of the approach.

### Recalibration of sea level projections

Theimpact of our revised ${+16.0}_{-5.6}^{+5.5}$ m Mid-Pliocene GMSL estimate on recent projections of future Antarctic contributions to sea level change is assessed by repeating the binary history matching procedure described in DeConto *et al.* (*1*). We focus on their RCP8.5 calculations because outputs are provided for the full, raw ensemble of input parameter values in this case (*n* = 196) whereas, for other emissions scenarios, the available ensembles have been trimmed using paleo–sea level and satellite constraints (*n* = 109). The constraints that are applied to calibrate their ice sheet model ensembles include the following: (i) observed ice mass loss between 1992 and 2017 from altimetry, gravimetry, and input-output methods [i.e., Ice sheet Mass Balance Inter-Comparison Exercise (IMBIE) (*84*); equivalent to GMSL change of +15 to 46 mm year^−1^]; (ii) estimated Antarctic contributions to LIG GMSL (4.6 ± 1.5 m); and (iii) estimated Antarctic contributions (16 ± 5 m) to Mid-Pliocene GMSL.

As in their analysis, we find that 163 models are consistent with the IMBIE constraint and 119 with both IMBIE and LIG target values. However, replacing their original estimate of the Antarctic contribution to Mid-Pliocene GMSL (16 ± 5 m) with our revised ${+9.8}_{-5.7}^{+5.6}$ m value substantially reduces the number of models consistent with all three constraints (15 versus 109). This reduction restricts the range of parameters controlling the MICI mechanism from 107 ± 54 m^−1^ year^2^ to 7 ± 7.5 m^−1^ year^2^ for the hydrofracturing prefactor, CALVLIQ, and 7.7 ± 3.3 km year^−1^ to 8.6 ± 2.6 km year^−1^ for the maximum calving rate parameter, VCLIFF. Consequently, although inclusion of both marine ice sheet instability and MICI mechanisms is required to fit the full range of revised constraints, the hydrofracturing component of MICI becomes a much smaller overall contributor.

## Supplementary Materials

### This PDF file includes:

Supplementary Text Figs. S1 to S11 Table S1 References
