# Supplementary material for: Geodynamically corrected Pliocene shoreline elevations in Australia consistent with midrange projections of Antarctic ice loss
Source: Sci Adv. 2023 Nov 17;9(46):eadg3035. doi: 10.1126/sciadv.adg3035 (PMC10656067; doi:10.1126/sciadv.adg3035)
Supplement: Supplementary file 1 — Supplementary Text Figs. S1 to S11 Table S1 References [file sciadv.adg3035_sm.pdf]

Science Advances
AAAS

# Supplementary Materials for

## Geodynamically corrected Pliocene shoreline elevations in Australia consistent with midrange projections of Antarctic ice loss

Fred D. Richards *et al.*

Corresponding author: Fred D. Richards, f.richards19@imperial.ac.uk

*Sci. Adv.* **9**, eadg3035 (2023)
DOI: 10.1126/sciadv.adg3035

**This PDF file includes:**

Supplementary Text
Figs. S1 to S11
Table S1
References

# Supplementary Text

## S1 Optimal models of present-day dynamic topography

Prior to investigating the impact of time-evolving dynamic topography on Australian palaeo sea-level data, we must first construct Earth models that satisfy all available geodynamic observables that relate to the present-day condition of the mantle. To do so, we adopted the approach of Richards *et al.* (34), in which mantle density structures are sought that optimise global fits to independent observations of dynamic topography, geoid height anomalies, and core-mantle boundary (CMB) excess ellipticity.

These density models are constructed in a near-identical manner to the input temperature fields used in our time-dependent simulations (see Section M2 above). Density above 400 km is derived from a modified version of the RHGW20 temperature and density model (61). Deeper than 300 km and outside dense basal sections of LLVPs, the same anelastically corrected and smoothed pyrolite look-up table is used to convert seismic velocities from five different $V_S$ tomographic models [LLNL-G3D-JPS (37); S40RTS (38); SAVANI (39); SEMUCB-WM1 (40); TX2011 (41)] into temperature and density. Mid-mantle $V_S$ structure is also high-pass filtered over the 1000–2000 km depth range and the two different density parameterisations are smoothly merged between 300 km and 400 km depth by taking their weighted average.

The principal difference between the density inputs used in our instantaneous and time-dependent mantle flow models is that, rather than setting a fixed density jump for compositionally distinct material within the basal LLVP layer, a range of non-pyrolitic compositional endmembers are explored. These include mid-ocean ridge basalt, chondrite-enriched basalt, and iron-enriched pyrolite [see Table 1 in Richards *et al.* (34)]. In cases where the composition is intermediate between pyrolite

**Table S1:** Optimal LLVP basal layer parameters for each combination of mantle density model and radial viscosity profile. Layer composition is assumed to be a mechanical mixture of pyrolite (89) and the quoted chemical component: MORB = mid-ocean ridge basalt (89); CEB = chondrite-enriched basalt (90); FSP = iron-enriched pyrolite (91, 92). $\chi_G$ = global geodynamic misfit [Equation 7 of Richards *et al.* (34)]; $r_A^{Aus}$ = Pearson's correlation coefficient for present-day dynamic topography and residual depth anomalies around Australian margins (see transect in Figure 1 of the main text and Figure **S1**); $\chi_A^{Aus}$ = misfit between present-day dynamic topography and residual depth anomalies around Australian margins [Equation 5 of Richards *et al.* (34)]. Models in bold = those shown in Figures 1, **S3** and **S4**; models in italics = those shown in Figure **S1**.

| Density | Viscosity | Thickness (km) | Composition | $\delta\rho_C$ (%) | $\chi_G$ | $r_A^{Aus}$ | $\chi_A^{Aus}$ |
|---|---|---|---|---|---|---|---|
| *LLNL-G3D-JPS* | *S10* | 100 | 90% CEB | 3.5 | 1.74 | 0.84 | 0.57 |
| LLNL-G3D-JPS | F10V1 | 100 | 100% FSP | 3.4 | 2.49 | 0.85 | 0.37 |
| **LLNL-G3D-JPS** | **F10V2** | 200 | 60% FSP | 2.0 | 2.79 | 0.84 | 0.35 |
| TX2011 | S10 | 210 | 53% CEB | 2.0 | 1.66 | 0.79 | 0.61 |
| TX2011 | F10V1 | 100 | 90% CEB | 3.5 | 1.20 | 0.81 | 0.41 |
| TX2011 | F10V2 | 100 | 100% CEB | 3.9 | 1.43 | 0.80 | 0.42 |
| SEMUCB-WM1 | S10 | 210 | 60% FSP | 2.0 | 2.00 | 0.76 | 1.08 |
| **SEMUCB-WM1** | **F10V1** | 200 | 60% FSP | 2.0 | 2.05 | 0.80 | 0.68 |
| *SEMUCB-WM1* | *F10V2* | 200 | 92% MORB | 2.0 | 2.50 | 0.79 | 0.67 |
| SAVANI | S10 | 110 | 90% MORB | 2.0 | 2.83 | 0.84 | 0.61 |
| SAVANI | F10V1 | 50 | 100% CEB | 3.9 | 1.69 | 0.83 | 0.46 |
| SAVANI | F10V2 | 100 | 100% MORB | 2.3 | 2.01 | 0.83 | 0.43 |
| S40RTS | S10 | 100 | 70% CEB | 2.7 | 1.66 | 0.80 | 0.85 |
| S40RTS | F10V1 | 100 | 80% FSP | 2.7 | 2.07 | 0.82 | 0.54 |
| S40RTS | F10V2 | 100 | 90% FSP | 3.0 | 2.46 | 0.81 | 0.55 |

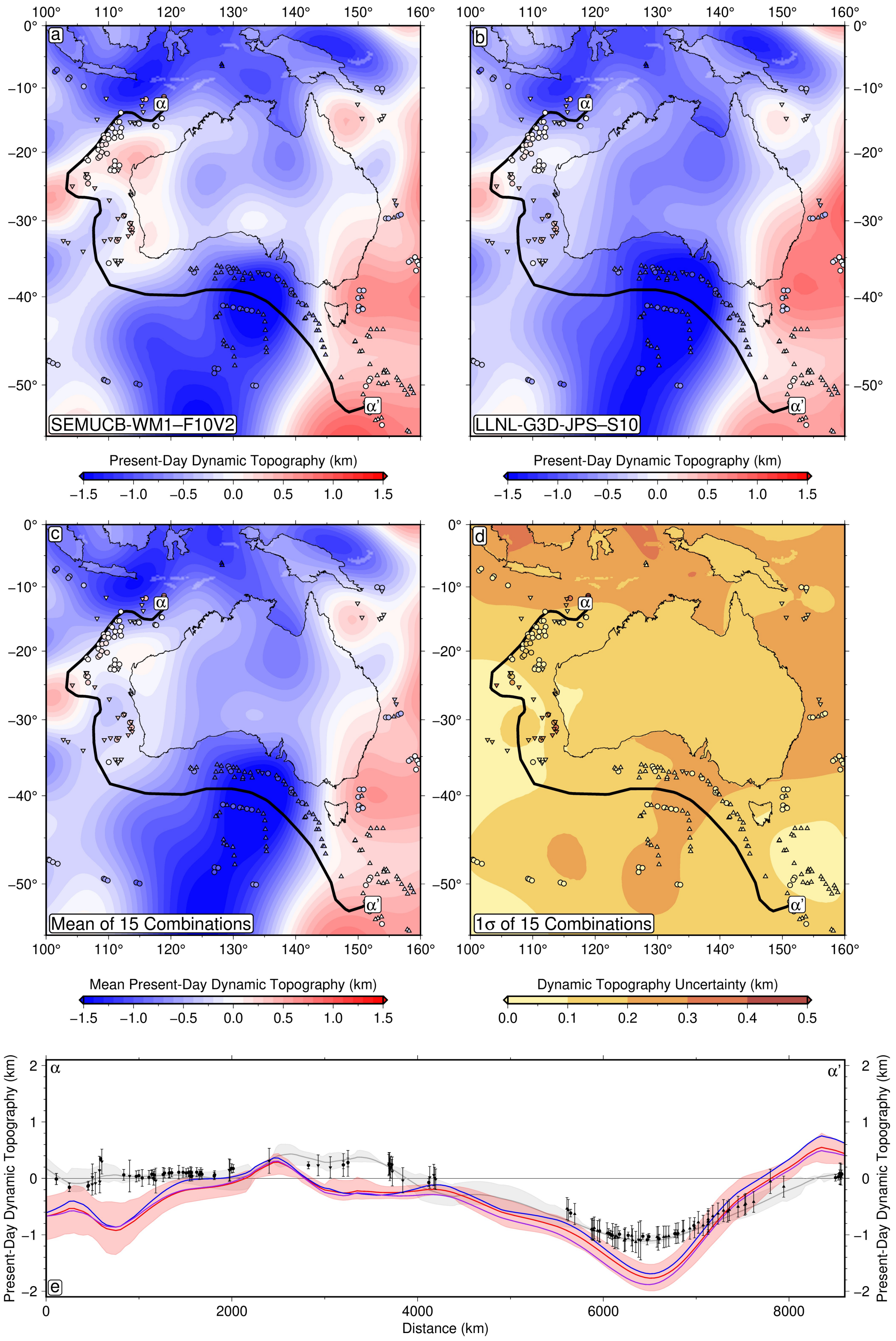


**Figure S1: Consistency between predicted present-day dynamic topography and measured oceanic residual depth anomalies.** (a) Predicted dynamic topography (water-loaded offshore, air-loaded onshore) for mantle density structure derived from SEMUCB-WM1 tomographic model (40) and the F10V2 mantle viscosity profile (36) expanded up to spherical harmonic degree, $l_{max} = 30$. Coloured circles/triangles = spot measurements of oceanic residual depth (86) (a common proxy for observed dynamic topography at present day); thick black line = location of transect shown in panel (e). (b) Same for LLNL-G3D-JPS tomographic model (37) and S10 mantle viscosity profile (35). (c) Mean dynamic topography across all 15 combinations of tomography and viscosity model. (d) Dynamic topography uncertainty ($1\sigma$) across all 15 models. (e) Predicted versus observed present-day dynamic topography along NW-to-SE transect. Red line/band = mean prediction with $1\sigma$ uncertainties; blue line = individual result for SEMUCB-WM1 and F10V2 combination; purple line = same for LLNL-G3D-JPS and S10; circles/triangles with error bars = spot measurements of residual depth and uncertainties (86); grey line = spherical harmonic fit to spot measurements ($l_{max} = 30$) with grey band representing range of values within a 500 km-wide swath perpendicular to transect.

and a given endmember, properties appropriate for a mechanical mixture of the two components are calculated using the Voigt-Reuss-Hill approximation to average the elastic moduli and generate an appropriate anelastically corrected and smoothed $V_S$ lookup table. For all models where LLVP basal layer composition is considered distinct from ambient mantle (i.e., the mantle is not 100% pyrolite), temperatures and densities are determined separately for the two components and then combined into a single array. For each candidate compositional endmember, density models are generated for enrichments of $[0, 10, ..., 100]$% and for dense basal layer thicknesses of 50 km and 100–900 km in 100 km increments. In each case, the mean density anomaly of the combined intra- and extra-LLVP regions is normalised to zero such that the mean density remains equal to PREM (93). By constructing these density structures from each of our five deep mantle $V_S$ models, we generate an ensemble of 505 mantle density inputs.

For each density input, we predict geoid undulations, surface dynamic topography, and CMB dynamic topography up to a maximum spherical harmonic degree, $l_{max} = 30$, using the instantaneous flow kernel methodology outlined in Corrieu *et al.* (94). This formulation accounts for the effects of both compressibility and self-gravitation [see Appendix B of Richards *et al.* (34) for details]. In each case, we assess the sensitivity of our results to Earth's uncertain viscosity structure by computing instantaneous mantle flow for three previously published radial profiles that are constrained by geoid, heat flow and glacial isostatic adjustment observations: S10 (35); F10V1; and F10V2 (36). For each pair of tomographic and radial viscosity inputs (15 in total), we then determine the optimal LLVP basal layer thickness and composition that fits observed geoid undulations, surface dynamic topography, and CMB excess ellipticity. Successful models must also exhibit an intrinsic density anomaly $\geq 2\%$ for chemically distinct material within the LLVP, which is the estimated lower bound for long-term preservation of chemical heterogeneity (95, 96, 97) [see Richards *et al.* (34) for misfit parameterisation].

The 15 best-fitting mantle density models obtained via this optimisation procedure yield LLVP basal layer thicknesses between 50 km and 210 km and 2.0–3.9% intrinsic compositional density differences. There is evidence for a negative trade-off between these two LLVP parameters. Nevertheless, for the purposes of this study, it is important to note that their predicted present-day dynamic topography fields remain consistent ($1\sigma \sim$190 m; Figure S1) and that they yield especially good agreement with oceanic residual depth anomalies around the margins of Australia (regional misfit is 25–65% smaller than the global average). These characteristics suggest that our present-day mantle density models are both robust and mutually compatible beneath this region, thereby validating their use in the time-dependent simulations that are central to this study.

## S2 Modelling time-dependent mantle flow

Having verified that our optimised mantle density models yield good agreement between predicted and observed present-day dynamic topography around Australia, we next incorporate these inputs into time-dependent simulations to hindcast (or 'retrodict') changes in dynamic topography since Mid-Pliocene times. We chose to implement these retrodictions using the convection code ASPECT, which required us to modify the $V_S$-to-density conversion methodology used in our instantaneous models to allow for reasonable computation times. First, rather than inferring lower mantle density from composition and temperature using Perple_X lookup tables, we instead apply a radial thermal expansivity profile. Our profile is determined by fitting a three-part linear model to laterally averaged depth-dependent expansivities obtained from our hybrid $V_S$-to-temperature conversion [i.e., the

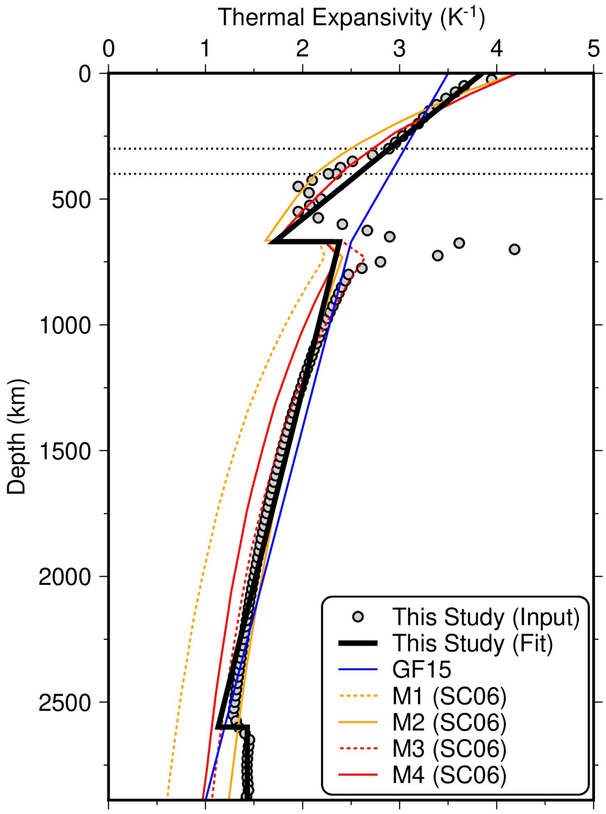


**Figure S2: Radial profiles of thermal expansivity.** Grey circles = modelled values based on anelastic parameterisation in upper 300 km, thermodynamic modelling of pyrolite with Perple_X and Stixrude & Lithgow-Bertelloni (68) database in lower 400 km, and a linear interpolation between the two from 300–400 km; dotted lines = 300 km and 400 km depth; solid line = 3-layer (0–670 km; 670–2600 km; and 2600 km-to-CMB) linear fit (N.B. values between 600–750 km are excluded during fitting due to strong effects of phase transitions that are unlikely to be tomographically resolved); coloured lines = reference profiles from other studies; GF15 = Glišović & Forte (76); SC06 = Steinberger & Calderwood (98).

Richards *et al.* (61) anelastic parameterisation shallower than 400 km, the Perple_X-derived pyrolite lookup table deeper than 300 km, and a weighted combination of the two between 300 km and 400 km depth; Figure **S2**]. Secondly, our ASPECT simulations are carried out under the Boussinesq approximation (i.e., they are incompressible). We therefore adjust our models by removing depth-dependent increases in temperature and density caused by adiabatic compression and by reducing the intrinsic compositional density jump for anomalous LLVP material to 0–132 kg m$^{-3}$ (i.e., 0–4% of the incompressible reference density, $\rho_0$ = 3300 kg m$^{-3}$). This adjustment minimises the difference between compressible and incompressible model predictions of dynamic topography, with compressible models yielding 10–15% higher dynamic topography at long wavelengths ($l$ = 1–5) and minimal offset at shorter wavelengths. These differences are negligible in the context of this study, since changes in dynamic topography since 3 Ma are dominated by short-wavelength signals driven by shallow mantle dynamics. Although compressibility has a larger effect on the predicted non-hydrostatic geoid, predicted rates of change exhibit minimal differences and, since radial surface displacement has a stronger impact on relative sea-level variation than perturbations to the equipotential, the impact on our sea-level predictions is modest (generally $< 0.1$ m).

Despite details of the density fields exhibiting substantial differences (in keeping with the variations between tomographic models that underpin them), we find that, in all cases, the long-wavelength pattern of mantle flow is dominated by near-vertical lower mantle downwelling beneath Australia

(Figures **S3**–**S4**). Similarly, shallow mantle upwellings are predicted to occur beneath Cape Range, Tasmania, the South Eastern Highlands, and Cape York in all models; however, the connection between these features and deep mantle structure is not clear in all cases (e.g., compare Figures **S3**d–e and Figures **S4**d–e beneath Cape Range and Tasmania). Despite substantial discrepancies in short-wavelength mid-mantle buoyancy structure, the pattern of predicted Mid-Pliocene-to-Recent dynamic topography change is consistent across our model ensemble, highlighting the dominant influence of the shallow mantle and the relative insensitivity of the vertical surface motions to deeper structure except at the longest wavelengths (Figures **S5**–**S7**).

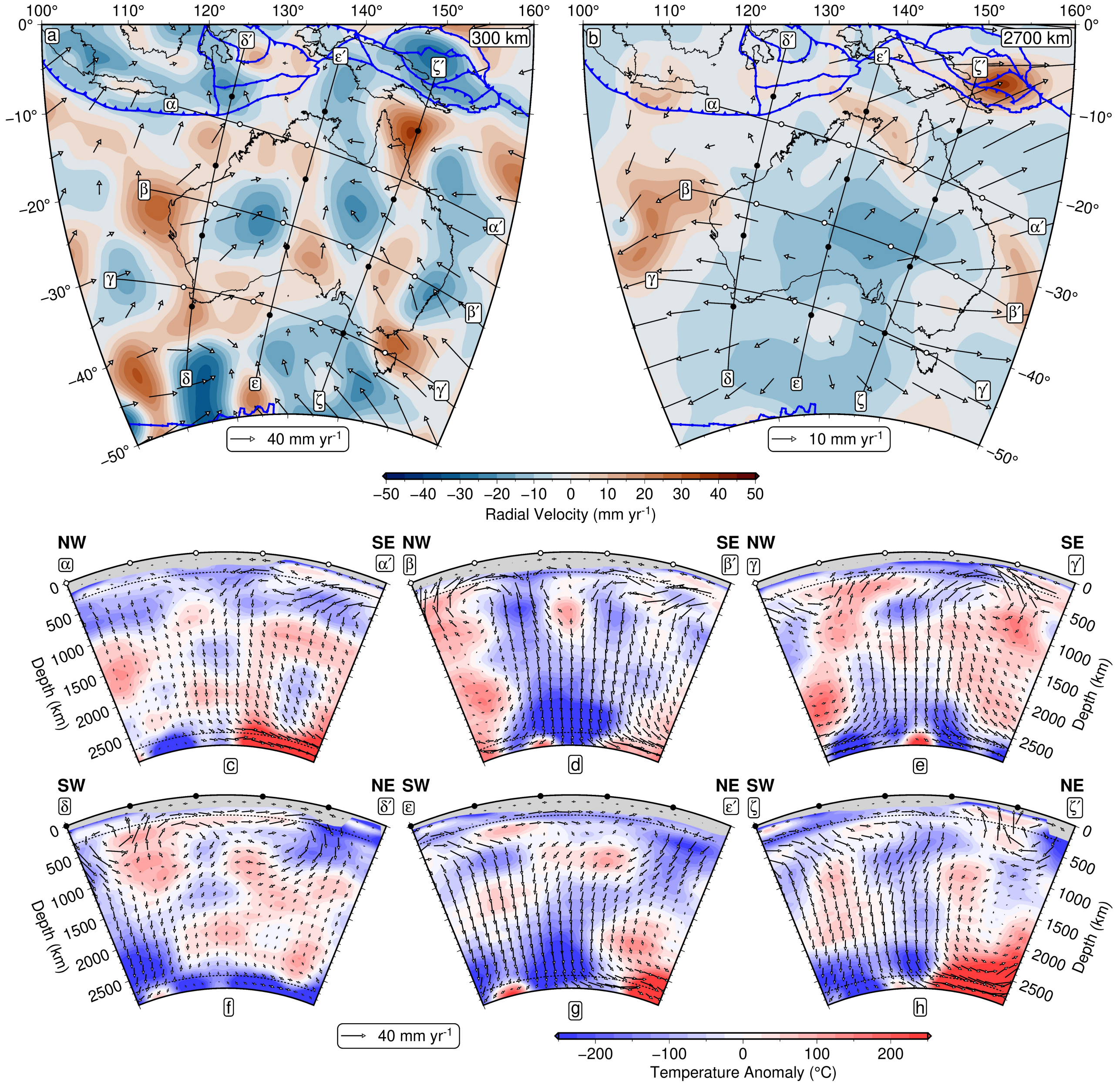


**Figure S3: Cross-sections through convection simulation based on SEMUCB-WM1 tomographic model (40) and F10V1 viscosity profile (36).** (a) Grid = radial component of present-day mantle flow at 300 km depth; arrows = tangential component of flow; blue lines = plate boundaries. (b) Same as panel (a), except at 2700 km. (c–h) Cross-sections along transects shown in panels (a–b). Temperature anomaly given by red-blue colourscale; arrows = flow velocity in plane of transect; grey region = lithosphere (defined using depth to 1200 °C isotherm).

Some systematic differences do, however, emerge in the long-wavelength pattern of dynamic to-

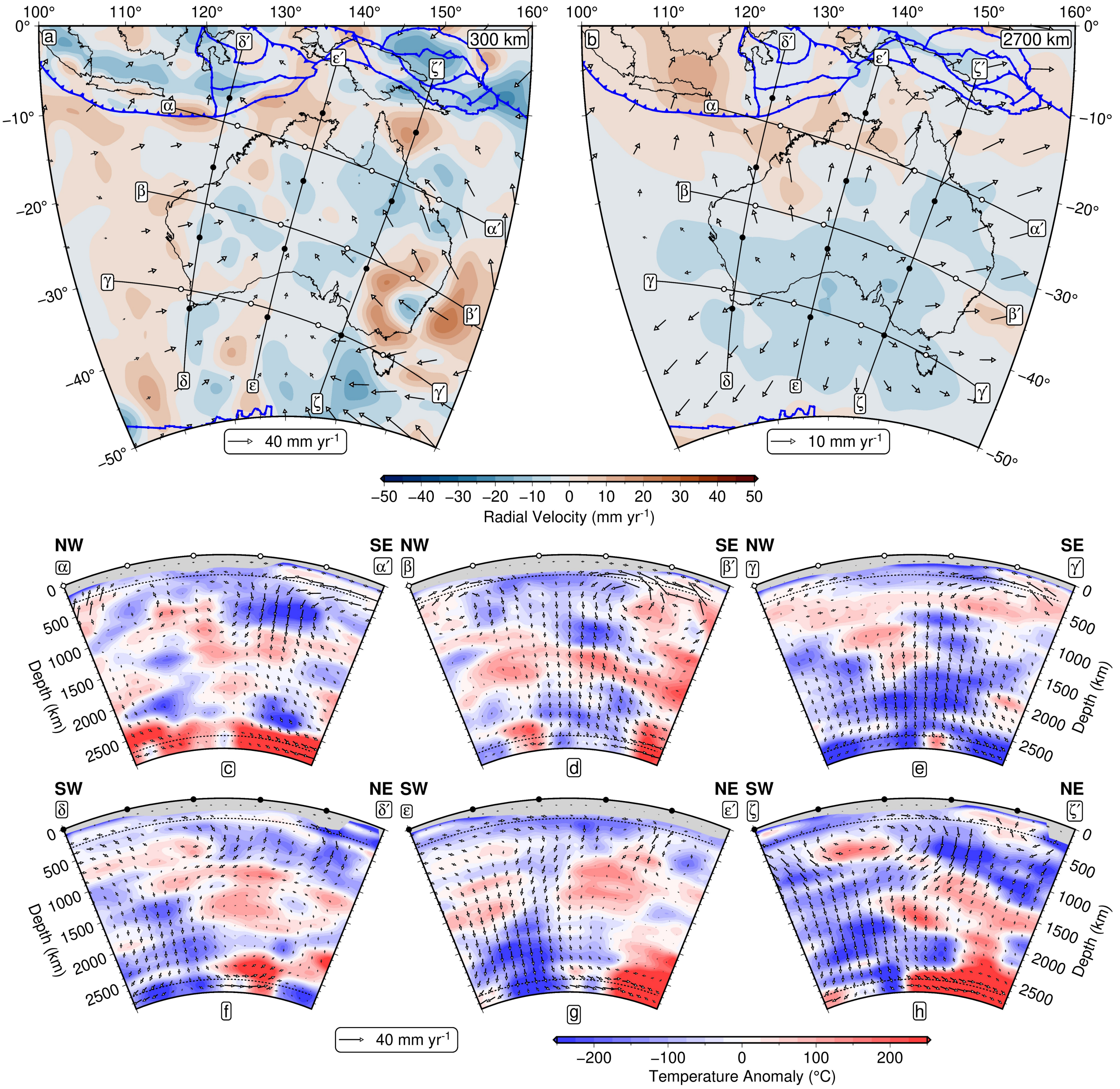


**Figure S4: Cross-sections through convection simulation based on LLNL-G3D-JPS tomographic model (37) and F10V2 viscosity profile (36).** Panels follow Figure **S3**.

pography change predicted by the different models. SEMUCB-WM1-based models feature stronger upwelling along the western Australian margin, leading to greater uplift at Cape Range and in the Perth Basin, while LLNL-G3D-JPS- and S40RTS-based models produce more pronounced uplift over the South Eastern Highlands (Figure **S5**). Models adopting the F10V1 viscosity profile yield the most uplift along the Eastern Highlands and relatively modest uplift along the western margin, with the reverse being true for S10-based counterparts.

Changes in the thickness and intrinsic density of the basal LLVP layer have a minor but detectable impact on predicted dynamic topography patterns, with thicker, denser endmembers generally producing less uplift in the southwest and more in the northeast (Figures **S6** and **S7**). This pattern is to be expected since the southwestern edge of the Pacific LLVP strikes approximately northwest-southeast beneath Cape York, with variations in intrinsic LLVP density therefore influencing the gradient in dynamic topography perpendicular to this boundary. Modifying the plate motion model from MORVEL

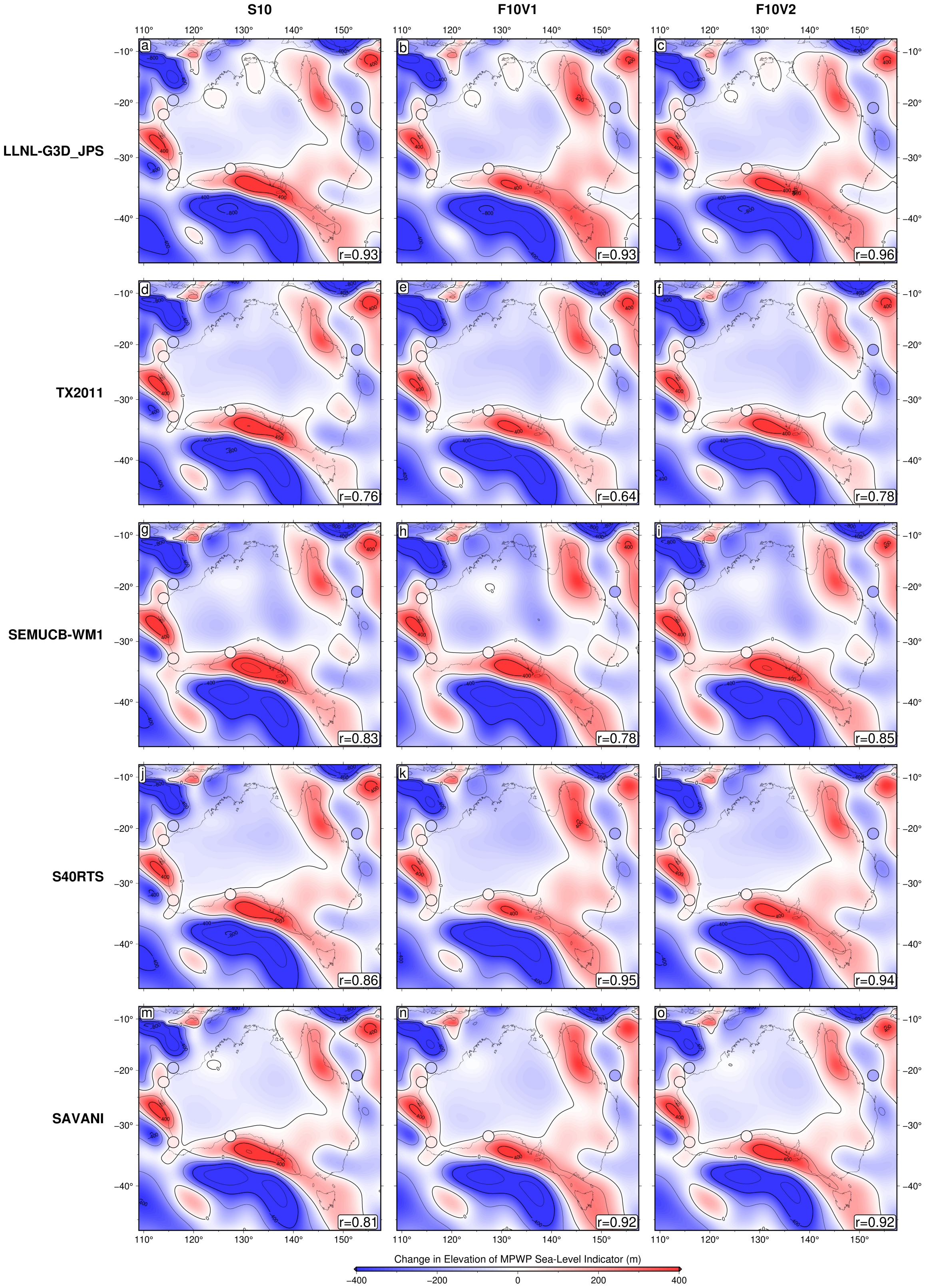


**Figure S5: Predicted Pliocene-to-Recent dynamic topography change as a function of tomography and viscosity input combination.** (a) Predicted change in elevation of Mid-Pliocene Warm Period sea-level markers for LLNL-G3D-JPS tomographic model and S10 viscosity profile. Circles = Mid-Pliocene median uncorrected GMSL estimates (i.e., present-day elevation + palaeo-water depth; Table 2 in main text); $r$ = correlation coefficient between predicted dynamic topography change and uncorrected GMSL estimates. (b) Same for LLNL-G3D-JPS and F10V1. (c) Same for LLNL-G3D-JPS and F10V2. (d–f) Same for TX2011. (g–i) Same for SEMUCB-WM1. (j–l) Same for S40RTS. (m–o) Same for SAVANI. Note that all models include a 100 km-thick, basal LLVP layer with $\Delta\rho_c = +2\%$ intrinsic density contrast. Plate motion is set by the MORVEL plate velocity model (77).

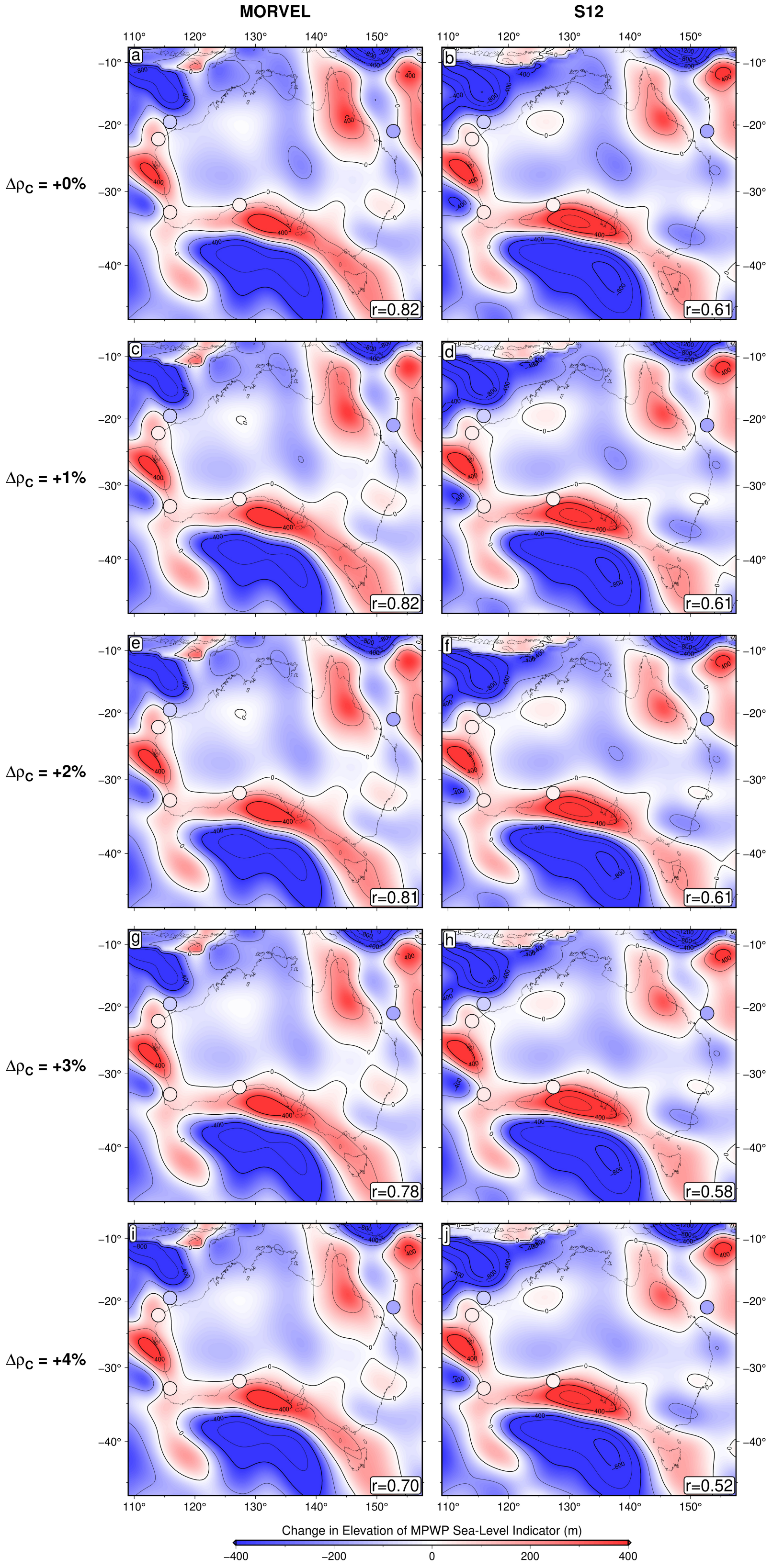


**Figure S6: Predicted Pliocene-to-Recent dynamic topography change as a function of intrinsic density contrast of basal LLVP layer and plate velocity model for the SEMUCB-WM1 tomographic model and F10V1 viscosity pairing.** (a) Predicted change in elevation of MPWP sea-level markers for purely thermal model ($\Delta\rho_C = 0\%$) and MORVEL plate velocity model (77). Circles = Mid-Pliocene median uncorrected GMSL estimates (i.e., present-day elevation + palaeo-water depth; Table 2 in main text). (b) Same for the S12 plate velocity model (78). (c–d) Same for a thermochemical model with $\Delta\rho_C = +1\%$. (e–f) Same for a thermochemical model with $\Delta\rho_C = +2\%$. (g–h) Same for a thermochemical model with $\Delta\rho_C = +3\%$. (i–j) Same for a thermochemical model with $\Delta\rho_C = +4\%$. Note that the basal LLVP layer is 200 km thick for all cases.

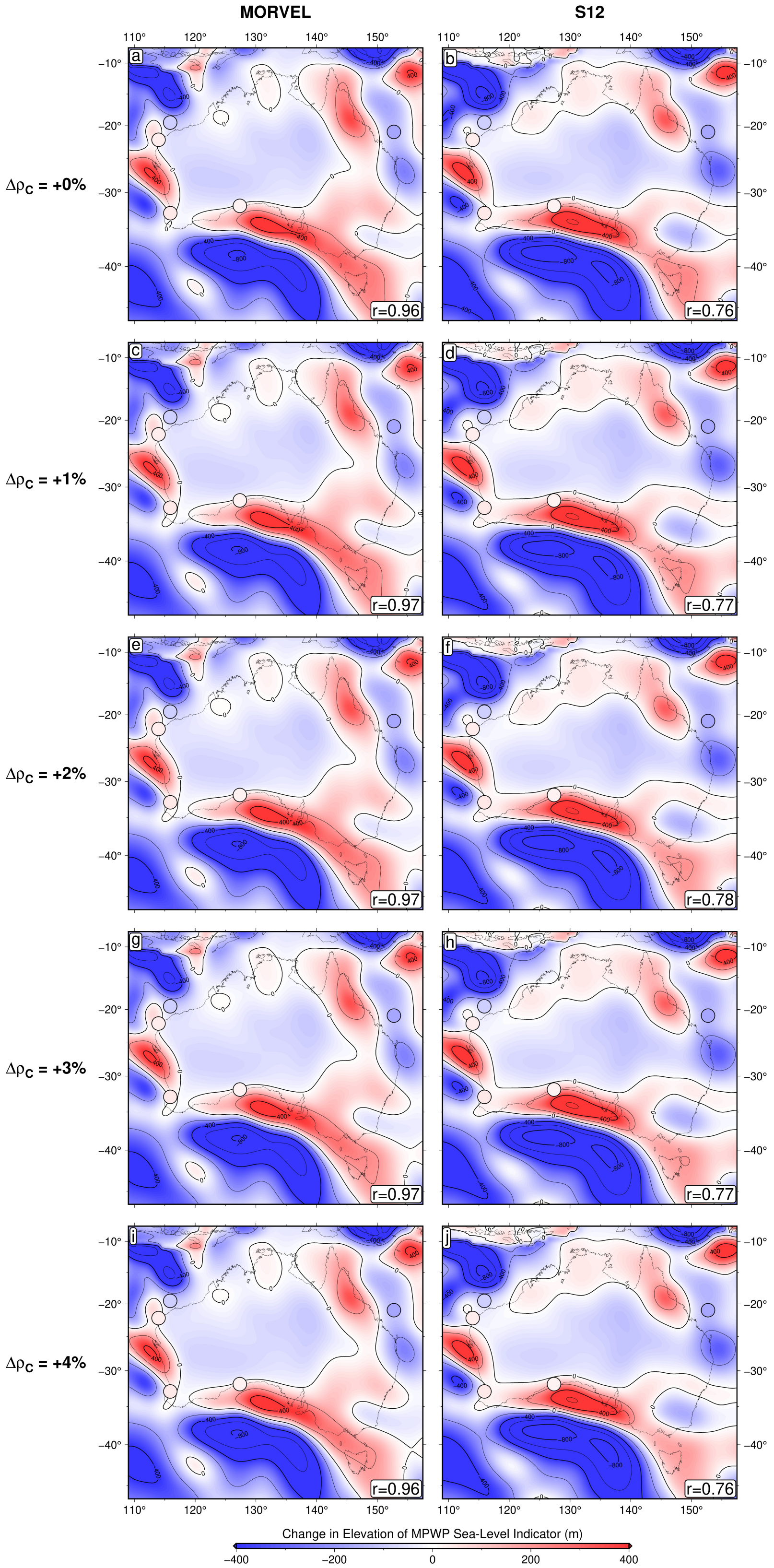


**Figure S7: Predicted Pliocene-to-Recent dynamic topography change as a function of intrinsic density contrast of basal LLVP layer and plate velocity model for the LLNL-G3D-JPS tomographic model and F10V2 viscosity pairing.** Panels follow Figure **S6**.

to S12 has a larger impact on predicted dynamic topography change, with the latter producing higher rates of uplift and subsidence (Figures **S6** and **S7**). Altering the plate motion model also induces substantial changes in the pattern of dynamic topography evolution; however, these differences depend strongly on the assumed viscosity and density model, reflecting associated changes in the direction and rate of flow in the shallow mantle.

Density models derived from S40RTS, LLNL-G3D-JPS, and SEMUCB-WM1 yield the best agreement with observed MPWP sea-level marker elevations, while the optimal radial viscosity profile covaries with input tomography and assumed LLVP structure. Best-fitting basal LLVP layer parameters combine relatively modest intrinsic density anomalies of 1–2% with upper bound layer thicknesses of ∼200 km. In all cases, significantly improved fit between predicted and observed sea-level marker elevations is obtained for the MORVEL plate motion model in comparison to S12 ($r$ is on average ∼0.2 higher; Figures **S6** and **S7**).

## S3 Modelling Pliocene-to-Recent GIA-induced sea-level change

Australia is in the far field of both the former Fennoscandian and Laurentian ice sheets, the present-day Greenland and West Antarctic Ice Sheets, and most marine-based sectors of the East Antarctic Ice Sheet. Thus, ongoing continental levering in response to the last deglaciation is expected to be the dominant process responsible for introducing geographic variability into GIA-related relative sea-level change around the continent. There are two main sources of uncertainty in our GIA models that must be considered – the ice history and the mantle viscosity structure.

The configuration of ice sheets at the end of the MPWP is uncertain and different assumptions introduce changes to the amplitude of predicted relative sea-level variations (Figure **S8**). In particular, ice history 'B' (MPWP ice configuration equivalent to that of the present day minus the Greenland and West Antarctic ice sheets) yields predicted sea-level marker elevations that are up to ∼70% more negative than those obtained with ice history 'A' (MPWP ice configuration identical to the present day). This difference arises due to additional continental levering associated with the rapid increase in ice volume at 2.95 Ma in history A, but has minimal impact on our compilation of sea-level markers since they are mostly located close to the coastlines where the net contribution of this effect is close to zero.

Differences in the assumed rheological structure of the mantle cause subtle modifications to the predicted pattern of marker elevation change (Figure **S8**). Switching from the 120p55 radial profile to 80CV2V2 increases the amplitude of both positive and negative deviations by up to 50%. This result reflects both an increase in levering amplitude associated with the thinner lithosphere in 80CV2V2 and an overall stronger GIA response to deglacial ice-mass change caused by the reduced lower mantle viscosity in this model.

Nonetheless, these models indicate that no plausible combination of ice sheet configuration or mantle rheological structure can be responsible for the observed spatial variability in MPWP sea-level marker elevations around Australia. The predicted amplitudes of elevation change are almost two orders of magnitude too small and the average correlation with the marker elevations is only $\bar{r} \sim -0.04$. Furthermore, the effect of introducing lateral viscosity variations into GIA predictions has been shown to be relatively minor around Australia (67). This factor suggests that our use of simple, purely radial Earth structure to explore GIA uncertainties is sufficient. Nevertheless, despite its limited ability to account for spatial variability in sea-level indicator elevations, correcting for GIA

processes does increase our final MPWP GMSL estimate by ∼4 m.

Although we do not account for East Antarctic ice loss in the full GIA calculations outlined above, we have quantified the potential impact of melting certain marine-based sectors on measured RSL at our MPWP sea-level marker sites. These tests simulate steady loss of ice from Prydz Bay (∼1.5 m GMSLE) and Wilkes Subglacial Basin (∼3 m GMSLE) over the course of 5 kyr using the same modelling framework as the full simulations and the `120p55` viscosity profile. We find that RSL deviates from GMSL by less than a metre at all five sites. This result indicates that our conclusions are not materially impacted by our omission of spatially variable sea-level signals that could be caused by Mid-Pliocene ice loss from different sectors of the East Antarctic Ice Sheet.

## S4 Bayesian Gaussian process-based inversion for MPWP GMSL

By framing estimation of MPWP GMSL as a Bayesian Gaussian process-based regression problem, we are able to constrain the posterior probability distribution of its value given: i) prior knowledge of its likely magnitude and temporal variation; ii) constraints on the age and palaeo-water depth of individual sea-level markers; and iii) our ensemble of GIA and dynamic topography predictions. This inference process permits GMSL solutions that produce temporal evolution consistent with the timescale and variance parameters of the underlying Gaussian process, while, at any individual timestep, model

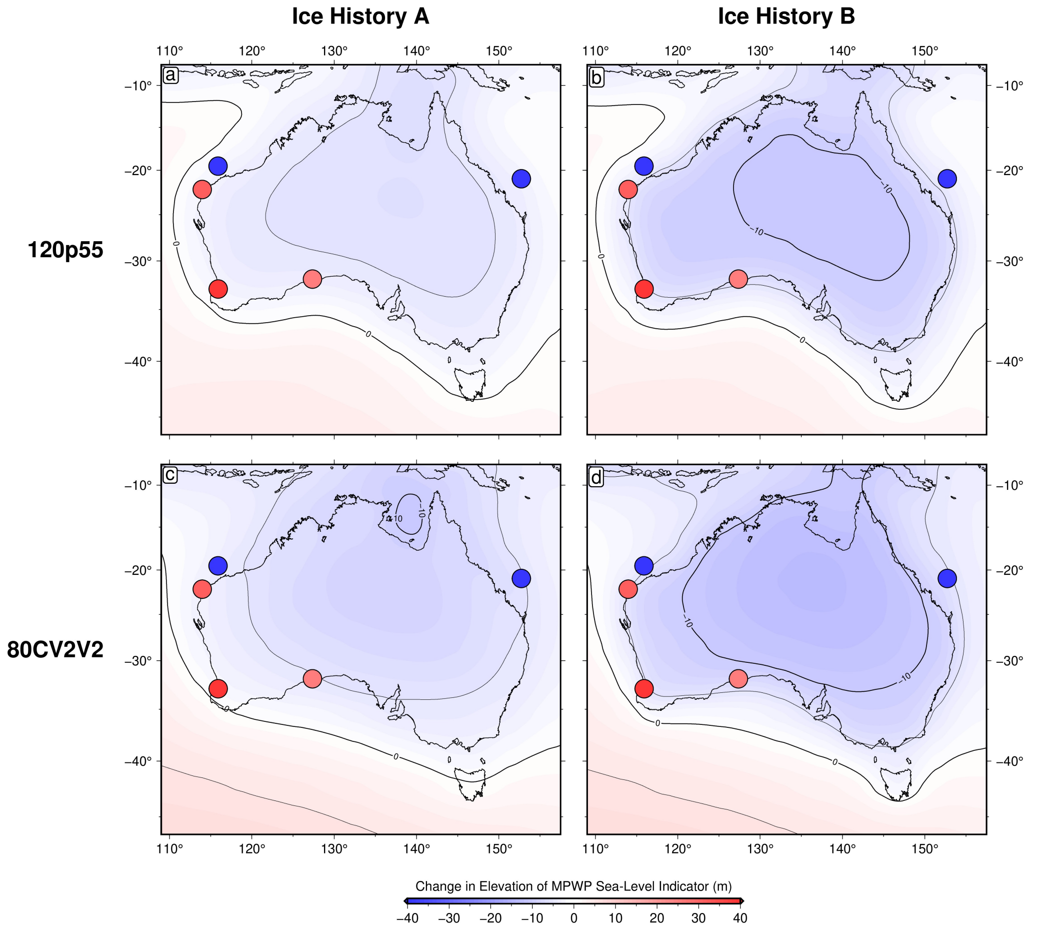


**Figure S8: Predicted GIA-induced change in MPWP sea-level marker elevations as a function of assumed mantle viscosity and ice history.** (a) Predicted change in elevation of MPWP sea-level markers for `120p55` viscosity profile and ice history A, in which MPWP ice volume is equivalent to that at the present day. (b) Same for `120p55` viscosity profile and ice history B, in which Greenland and West Antarctic ice sheets are absent during the MPWP (∼+14 m GMSLE compared to present-day). (c–d) Same for `80CV2V2` viscosity profile.

realisations that minimise spatial variability in sea level are naturally favoured. Consequently, the resulting posterior distributions of individual input parameters yield useful information concerning which density and viscosity models best account for MPWP sea-level indicator elevations. They therefore provide new constraints on Earth's internal structure as well as some quantification of related uncertainties in estimated GMSL.

The posterior distribution for GIA-induced elevation changes closely resembles the prior in terms of both median value and uncertainty range. This result is to be expected since the elevation changes are much smaller than those associated with both the dynamic topography correction and the observational data spread (Figure **S9**). GIA-induced elevation changes are least well-constrained in the interior of the continent (Figure **S9**d). The maximum *a posteriori* (MAP) estimates are $\sim 0.1$ for the GIA viscosity profile index and $\sim 11$ m GMSLE for the ice history, indicating a slight preference for the `120p55` viscosity model and an MPWP ice sheet configuration in which large portions of the Greenland and West Antarctic ice sheets were initially absent (Figures **S10**l and **S10**m). Nevertheless, uncertainties on these two parameters are sufficiently large that no firm conclusions can be drawn from this analysis.

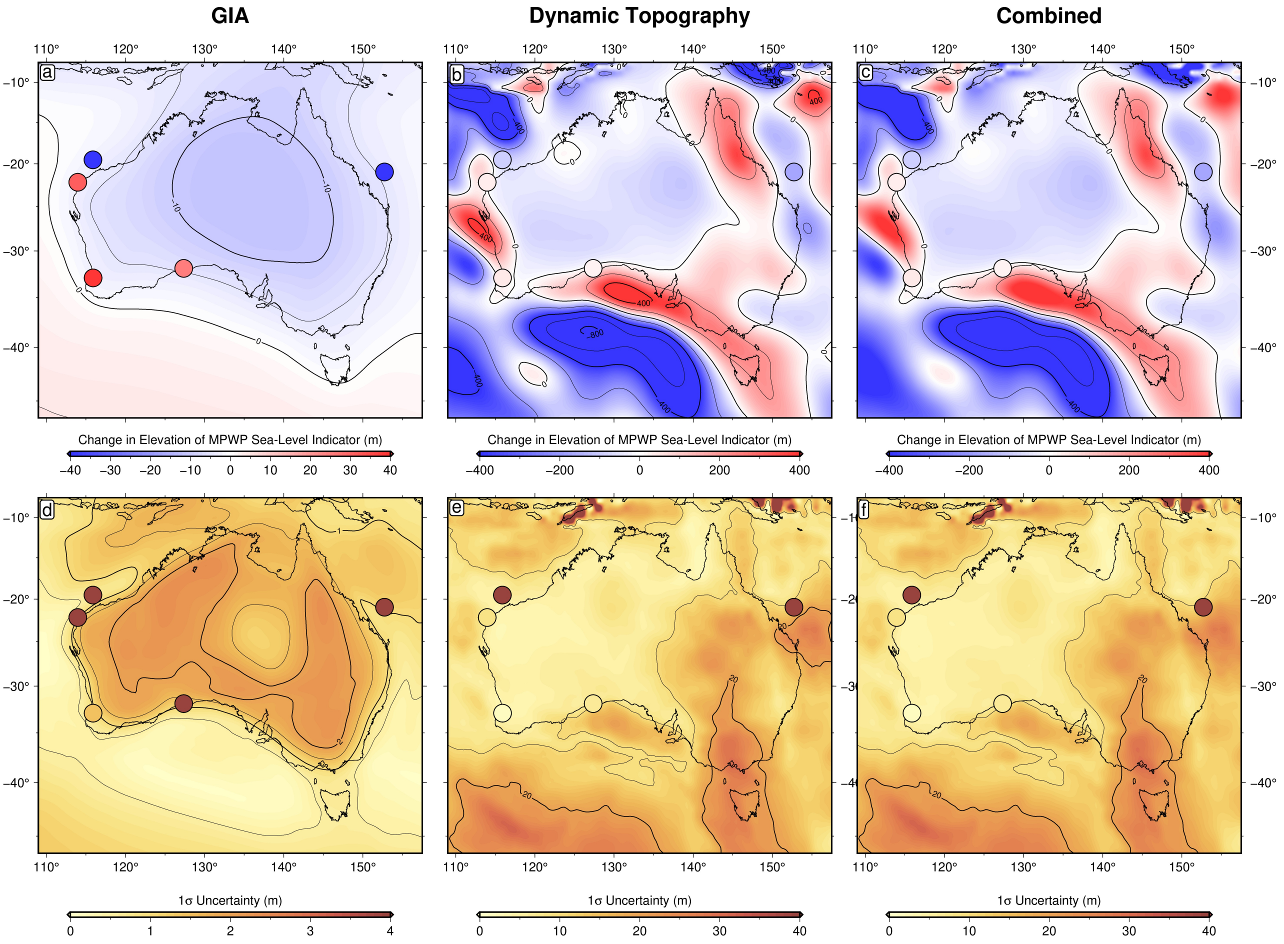


**Figure S9: Comparison of posterior GIA and dynamic topography elevation change predictions.** (a) Posterior median for predicted GIA. Circles = Mid-Pliocene median uncorrected GMSL estimates (i.e., present-day elevation + palaeo-water depth; Table 2 in main text). (b) Same for dynamic topography. (c) Same for combined prediction. (d) Posterior uncertainty for predicted GIA (i.e., half of the difference between 16$^{\text{th}}$ and 84$^{\text{th}}$ percentile values). Circles = uncertainty in Mid-Pliocene GMSL estimates (i.e., combined elevation and palaeo-water depth uncertainty; Table 2 in main text). (e) Same for dynamic topography. (f) Same for combined prediction. Note change in colour bar ranges between GIA and dynamic topography panels due to substantially lower amplitudes in the former.

In contrast, the posterior distribution for predicted elevation change due to dynamic topography is significantly different to the prior distribution. The median dynamic topography change is closest to that expected for a model based on the S40RTS tomography model, the F10V1 radial viscosity profile, and the MORVEL plate motion model, with an LLVP dense basal layer thickness of $\sim 100$ km and intrinsic density contrast of $\Delta\rho_C \sim +2.0\%$ (Figure **S9**). Spatial variations in elevation change uncertainty are variable, but are particularly pronounced in southeastern Australia and close to the Australian-Antarctic Discordance where there are substantial differences in the shallow to mid-mantle $V_S$ structure of the input tomographic models (Figure **S9**e). Dynamic topography dominates over GIA when the two fields are combined, as demonstrated by the similarity of panels b versus c, and e versus f in Figure **S9**.

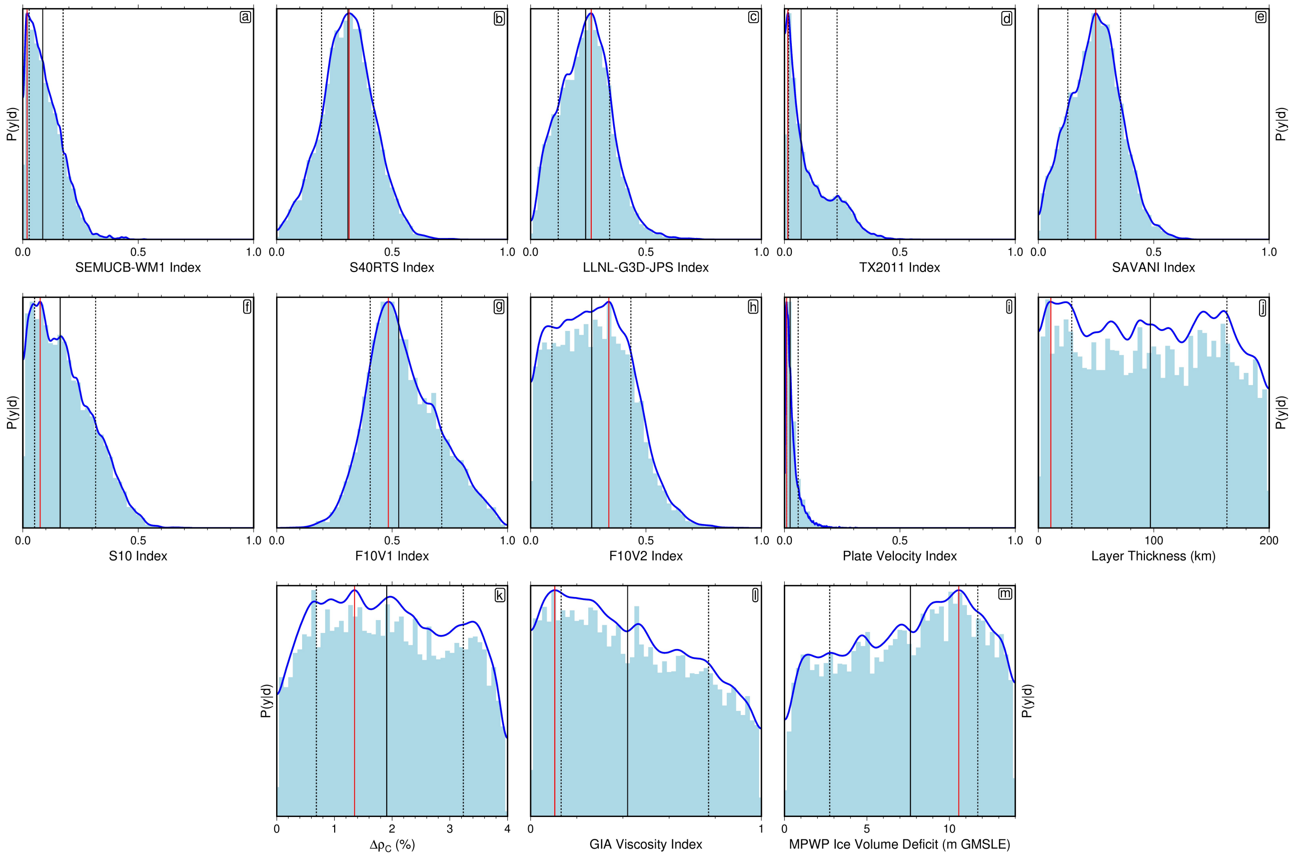


**Figure S10: Optimal model parameters.** (a–m) Marginal posterior probability distributions of model parameters. Blue bars = sample frequency; blue line = kernel density estimate; red line = estimated maximum *a posteriori* probability value (MAP); solid black line = median value; dotted black lines = 16$^{th}$ and 84$^{th}$ percentiles. Note that parameter values sum to unity for both the five tomographic model indices (SEMUCB-WM1/S40RTS/LLNL-G3D-JPS/TX2011/SAVANI) and three dynamic topography viscosity profile indices (S10/F10V1/F10V2). For the plate velocity model index, 0 = MORVEL (77), 1 = S12 (78); while, 0 = 120p55, 1 = 80CV2V2 for the GIA viscosity profile index. MPWP ice volume deficits are registered relative to the present-day (i.e., 0 m GMSLE = same as present day; 14 m GMSLE = additional absence of Greenland and West Antarctic ice sheets).

Posterior distributions for individual dynamic topography parameters and parameter pairs suggest that observed sea-level marker elevations are most consistent with mantle flow models based predominantly on S40RTS (MAP index value $\sim 0.3$), with modest contributions from LLNL-G3D-JPS and/or SAVANI (MAP values $\sim 0.2$ in both cases; Figures **S10**a–e and **S11**a–j and ca). Simulations with radial viscosity parameterisations similar to F10V1 are clearly optimal (MAP index value $\sim 0.5$); however, composites of F10V1 and the other profiles investigated here, F10V2 and S10, also yield high posterior

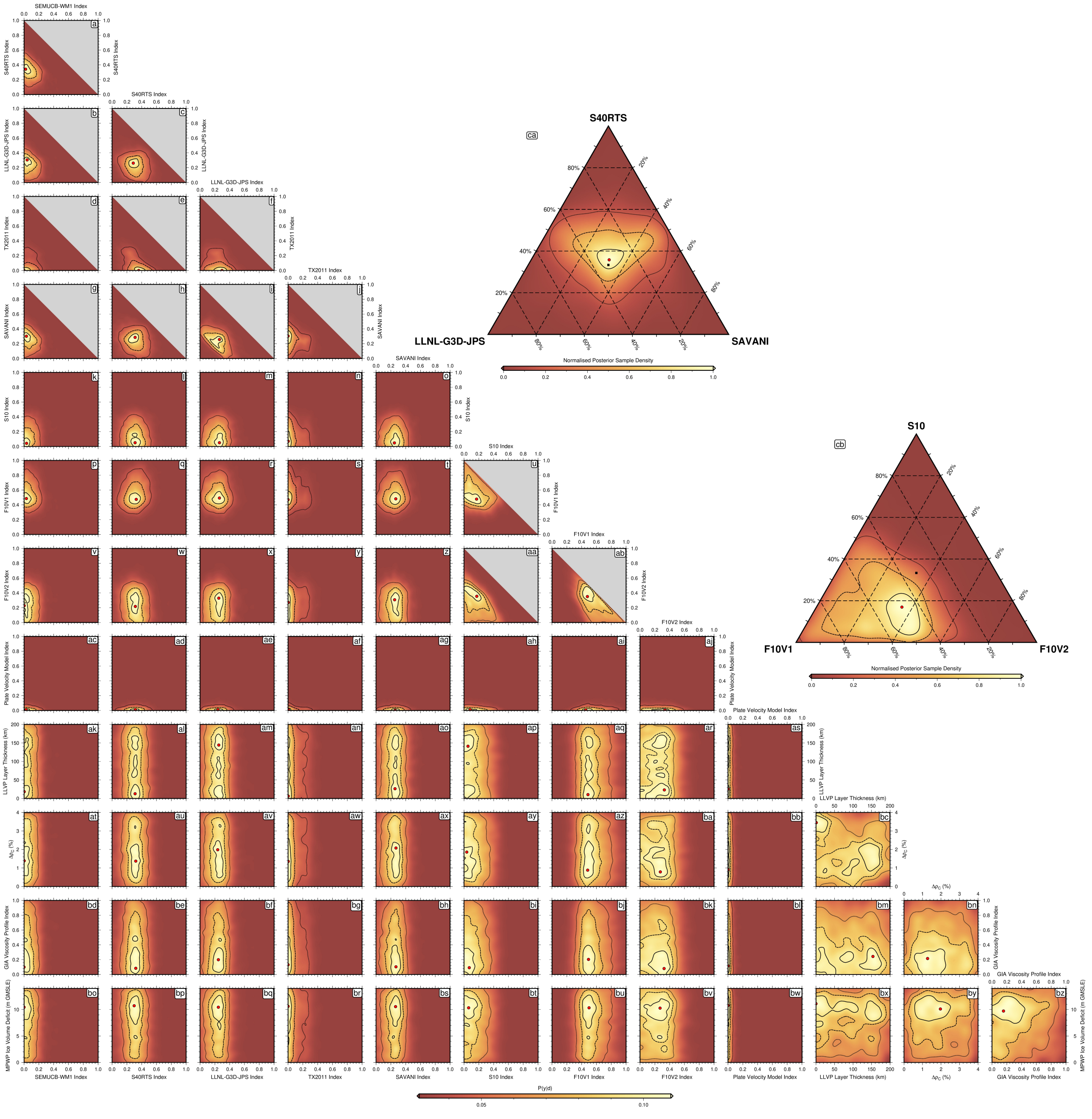


**Figure S11: Trade-offs between model parameters.** (a–bz) Joint posterior probability distributions (kernel density estimates) of model parameters. Solid contour = 84$^{\text{th}}$ percentile; dashed contour = median value; dotted black lines = 16$^{\text{th}}$ percentile; red circle = maximum *a posteriori* (MAP) value. Gray shading = region of parameter space that cannot be sampled since neither sum of tomographic model indices nor sum of dynamic topography viscosity profile indices can exceed unity. (ca) Posterior sample density (normalised so maximum value is unity) as a function of three tomographic model indices with highest MAP values (S40RTS, SAVANI, and LLNL-G3D-JPS). (cb) Same for indices of viscosity profiles used in dynamic topographic predictions.

probability (Figures **S10**f–h and **S11**u, aa, ab, and cb). The index for the plate motion model has the most tightly constrained posterior distribution of all input parameters, further demonstrating that the MORVEL plate motion model produces better agreement with our observations than the S12 model (Figures **S10**i and **S11**ac–aj, as, bb, bi, and bw). The posterior distributions for the two parameters controlling the properties of the dense basal layer within the LLVP (i.e., layer thickness and $\Delta\rho_C$) are less well constrained and exhibit some negative trade-off, with thick, less dense layers yielding similar posterior probability to thin, denser layers (Figure **S11**bc). These distributions are also skewed towards the lower bound of each parameter, with MAP estimates of $\sim$ 25 km for layer thickness and $\sim +1.5\%$ for $\Delta\rho_C$, but median estimates of $\sim$ 100 km and $\sim +2\%$, respectively (Figure **S10**j–k).

It is important to note that parameters controlling the dynamic topography predictions do not trade off with those underpinning the GIA calculations (Figure **S11**). This result suggests that, in future work, it may be possible to disentangle the two contributions even when they have more comparable magnitudes, for example, in near-field locations or over shorter timescales (e.g., since the Last Interglacial). Although most of the dynamic topography parameters have well constrained posteriors, larger uncertainties for LLVP-related values likely reflect limited impact of the Pacific LLVP on mantle flow patterns beneath Australia. These parameters might be better determined by applying our analysis in other regions, such as Southern Africa, where LLVP properties will have a much stronger effect on the rate and pattern of dynamic topography change.

**REFERENCES AND NOTES**

1. R. M. DeConto, D. Pollard, R. B. Alley, I. Velicogna, E. Gasson, N. Gomez, S. Sadai, A. Condron, D. M. Gilford, E. L. Ashe, R. E. Kopp, L. Dawei, A. Dutton, The Paris Climate Agreement and future sea-level rise from Antarctica. *Nature* **593**, 83–89 (2021).
2. A. M. Haywood, D. Hill, A. Dolan, B. L. Otto-Bliesner, F. Bragg, W.-L. Chan, M. A. Chandler, C. Contoux, H. J. Dowsett, A. Jost, Y. Kamae, G. Lohmann, D. J. Lunt, A. Abe-Ouchi, S. J. Pickering, G. Ramstein, N. A. Rosenbloom, U. Salzmann, L. Sohl, C. Stepanek, H. Ueda, Q. Yan, Z. Zhang, Large-scale features of Pliocene climate: Results from the Pliocene Model Intercomparison Project. *Clim. Past* **9**, 191–209 (2013).
3. E. De La Vega, T. B. Chalk, P. A. Wilson, R. P. Bysani, G. L. Foster, Atmospheric $CO_2$ during the Mid-Piacenzian Warm Period and the M2 glaciation. *Sci. Rep.* **10**, 11002 (2020).
4. M. E. Raymo, J. X. Mitrovica, M. J. O'Leary, R. M. DeConto, P. J. Hearty, Departures from eustasy in Pliocene sea-level records. *Nat. Geosci.* **4**, 328–332 (2011).
5. K. D. Burke, J. W. Williams, M. A. Chandler, A. M. Haywood, D. J. Lunt, B. L. Otto-Bliesner, Pliocene and Eocene provide best analogs for near-future climates. *Proc. Natl. Acad. Sci.* **115**, 13288–13293 (2018).
6. R. M. DeConto, D. Pollard, Contribution of Antarctica to past and future sea-level rise. *Nature* **531**, 591–597 (2016).
7. D. Pollard, R. M. DeConto, Modelling West Antarctic ice sheet growth and collapse through the past five million years. *Nature* **458**, 329–332 (2009).
8. S. Koenig, A. Dolan, B. De Boer, E. Stone, D. Hill, R. DeConto, A. Abe-Ouchi, D. Lunt, D. Pollard, A. Quiquet, F. Saito, J. Savage, R. S. W. van de Wal, Ice sheet model dependency of the simulated Greenland Ice Sheet in the mid-Pliocene. *Clim. Past* **11**, 369–381 (2015).
9. M. E. Raymo, R. Kozdon, D. Evans, L. Lisiecki, H. L. Ford, The accuracy of mid-Pliocene $\delta^{18}$O-based ice volume and sea level reconstructions. *Earth Sci. Rev.* **177**, 291–302 (2018).
10. G. S. Dwyer, M. A. Chandler, Mid-Pliocene sea level and continental ice volume based on coupled benthic Mg/Ca palaeotemperatures and oxygen isotopes. *Philos. Trans. A. Math. Phys. Eng. Sci.* **367**, 157–168 (2009).
11. E. Rohling, G. L. Foster, K. Grant, G. Marino, A. Roberts, M. E. Tamisiea, F. Williams, Sea-level and deep-sea-temperature variability over the past 5.3 million years. *Nature* **508**, 477–482 (2014).

12. K. G. Miller, J. V. Browning, W. J. Schmelz, R. E. Kopp, G. S. Mountain, J. D. Wright, Cenozoic sea-level and cryospheric evolution from deep-sea geochemical and continental margin records. *Sci. Adv.* **6**, eaaz1346 (2020).
13. A. Rovere, M. E. Raymo, J. Mitrovica, P. J. Hearty, M. O'Leary, J. Inglis, The Mid-Pliocene sea-level conundrum: Glacial isostasy, eustasy and dynamic topography. *Earth Planet. Sci. Lett.* **387**, 27–33 (2014).
14. B. P. Horton, R. E. Kopp, A. J. Garner, C. C. Hay, N. S. Khan, K. Roy, T. A. Shaw, Mapping sea-level change in time, space, and probability. *Annu. Rev. Env. Resour.* **43**, 481–521 (2018).
15. O. A. Dumitru, J. Austermann, V. J. Polyak, J. J. Fornós, Y. Asmerom, J. Ginés, A. Ginés, B. P. Onac, Constraints on global mean sea level during Pliocene warmth. *Nature* **574**, 233–236 (2019).
16. B. R. Wardlaw, T. M. Quinn, The record of Pliocene sea-level change at Enewetak Atoll. *Quat. Sci. Rev.* **10**, 247–258 (1991).
17. A. Rovere, P. J. Hearty, J. Austermann, J. Mitrovica, J. Gale, R. Moucha, A. Forte, M. Raymo, Mid-Pliocene shorelines of the US Atlantic Coastal Plain—An improved elevation database with comparison to Earth model predictions. *Earth Sci. Rev.* **145**, 117–131 (2015).
18. G. Grant, T. Naish, G. Dunbar, P. Stocchi, M. Kominz, P. J. Kamp, C. Tapia, R. McKay, R. Levy, M. Patterson, The amplitude and origin of sea-level variability during the Pliocene epoch. *Nature* **574**, 237–241 (2019).
19. P. Hearty, A. Rovere, M. Sandstrom, M. O'Leary, D. Roberts, M. E. Raymo, Pliocene-Pleistocene stratigraphy and sea-level estimates, Republic of South Africa with implications for a 400 ppmv $C_2$ world. *Paleoceanogr. Paleoclimatol.* **35**, e2019PA003835 (2020).
20. S. E. Williams, J. M. Whittaker, J. A. Halpin, R. D. Müller, Australian-Antarctic breakup and seafloor spreading: Balancing geological and geophysical constraints. *Earth Sci. Rev.* **188**, 41–58 (2019).X
21. M. Sandiford, Neotectonics of southeastern Australia: Linking the Quaternary faulting record with seismicity and in situ stress. *Evolution and Dynamics of the Australian Plate* **372**, 107–119 (2003).
22. A. M. Heimsath, J. Chappell, K. Fifield, Eroding Australia: Rates and processes from Bega Valley to Arnhem Land. *Geol. Soc. Lond. Spec. Publ.* **346**, 225–241 (2010).
23. R. Moucha, G. A. Ruetenik, Interplay between dynamic topography and flexure along the U.S. Atlantic passive margin: Insights from landscape evolution modeling. *Global Planet. Change* **149**, 72–78 (2017).

24. J. Mitrovica, G. Milne, On the origin of late Holocene sea-level highstands within equatorial ocean basins. *Quat. Sci. Rev.* **21**, 2179–2190 (2002).
25. L. B. Collins, J. L. Baxter, Heavy mineral-bearing strandline deposits associated with high-energy beach environments, southern Perth Basin, Western Australia. *Journal of the Geological Society of Australia* **31**, 287–292 (1984).
26. R. D. Gee, Landscape evolution and Cenozoic sea-levels of the Geographe Bay hinterland, southwestern Australia. *J. R. Soc. West. Aust.* **105** 1–19 (2022).
27. M. R. Sandstrom, M. J. O'Leary, M. Barham, Y. Cai, E. T. Rasbury, K. M. Wooton, M. E. Raymo, Age constraints on surface deformation recorded by fossil shorelines at Cape Range, Western Australia. GSA Bulletin (2020).
28. K. Czarnota, M. Hoggard, N. White, J. Winterbourne, Spatial and temporal patterns of Cenozoic dynamic topography around Australia. *Geochem. Geophys. Geosyst.* **14**, 634–658 (2013).
29. L. DiCaprio, R. D. Müller, M. Gurnis, A dynamic process for drowning carbonate reefs on the northeastern Australian margin. *Geology* **38**, 11–14 (2010).
30. M. Sandiford, The tilting continent: A new constraint on the dynamic topographic field from Australia. *Earth Planet. Sci. Lett.* **261**, 152–163 (2007).
31. K. Czarnota, G. Roberts, N. White, S. Fishwick, Spatial and temporal patterns of Australian dynamic topography from river profile modeling. *J. Geophys. Res. Solid Earth* **119**, 1384–1424 (2014).
32. J. Engel, J. Woodhead, J. Hellstrom, S. White, N. White, H. Green, Using speleothems to constrain late Cenozoic uplift rates in karst terranes. *Geology* **48**, 755–760 (2020).
33. P. Ball, K. Czarnota, N. White, M. Klöcking, D. Davies, Thermal structure of eastern Australia's upper mantle and its relationship to Cenozoic volcanic activity and dynamic topography. *Geochem. Geophys. Geosyst.* **22**, e2021GC009717 (2021).
34. F. D. Richards, M. J. Hoggard, S. Ghelichkhan, P. Koelemeijer, H. C. Lau, Geodynamic, geodetic, and seismic constraints favour deflated and dense-cored LLVPs. *Earth Planet. Sci. Lett.* **602**, 117964 (2023).
35. B. Steinberger, S. C. Werner, T. H. Torsvik, Deep versus shallow origin of gravity anomalies, topography and volcanism on Earth, Venus and Mars, *Icarus* **207**, 564–577 (2010).
36. A. M. Forte, S. Quéré, R. Moucha, N. A. Simmons, S. P. Grand, J. X. Mitrovica, D. B. Rowley, Joint seismic-geodynamic-mineral physical modelling of African geodynamics: A reconciliation of

deep-mantle convection with surface geophysical constraints. *Earth Planet. Sci. Lett.* **295**, 329–341 (2010).

37. N. Simmons, S. Myers, G. Johannesson, E. Matzel, S. Grand, Evidence for long-lived subduction of an ancient tectonic plate beneath the southern Indian Ocean. *Geophys. Res. Lett.* **42**, 9270–9278 (2015).
38. J. Ritsema, A. Deuss, H. J. Van Heijst, J. H. Woodhouse, S40RTS: A degree-40 shear-velocity model for the mantle from new Rayleigh wave dispersion, teleseismic traveltime and normal-mode splitting function measurements. *Geophys. J. Int.* **184**, 1223–1236 (2011).
39. L. Auer, L. Boschi, T. Becker, T. Nissen-Meyer, D. Giardini, Savani: A variable resolution whole-mantle model of anisotropic shear velocity variations based on multiple data sets. *J. Geophys. Res. Solid Earth* **119**, 3006–3034 (2014).
40. S. W. French, B. Romanowicz, Broad plumes rooted at the base of the Earth's mantle beneath major hotspots. *Nature* **525**, 95–99 (2015).
41. S. P. Grand, Mantle shear-wave tomography and the fate of subducted slabs. *Philos. Trans. A. Math. Phys. Eng. Sci.* **360**, 2475–2491 (2002).
42. M. Kronbichler, T. Heister, W. Bangerth, High accuracy mantle convection simulation through modern numerical methods. *Geophys. J. Int.* **191**, 12–29 (2012).
43. T. Heister, J. Dannberg, R. Gassmöller, W. Bangerth, High accuracy mantle convection simulation through modern numerical methods–II: Realistic models and problems. *Geophys. J. Int.* **210**, 833–851 (2017).
44. J. Austermann, J. Mitrovica, Calculating gravitationally self-consistent sea level changes driven by dynamic topography. *Geophys. J. Int.* **203**, 1909–1922 (2015).
45. B. Steinberger, Topography caused by mantle density variations: Observation-based estimates and models derived from tomography and lithosphere thickness. *Geophys. J. Int.* **205**, 604–621 (2016).
46. T. Duvernay, D. R. Davies, C. R. Mathews, A. H. Gibson, S. C. Kramer, Linking intraplate volcanism to lithospheric structure and asthenospheric flow. *Geochem. Geophys. Geosyst.* **22**, e2021GC009953 (2021).
47. J. A. Dickinson, M. W. Wallace, G. R. Holdgate, S. J. Gallagher, L. Thomas, Origin and timing of the Miocene-Pliocene unconformity in southeast Australia. *J. Sediment. Res.* **72**, 288–303 (2002).

48. D. B. Rowley, A. M. Forte, R. Moucha, J. X. Mitrovica, N. A. Simmons, S. P. Grand, Dynamic topography change of the eastern United States since 3 million years ago. *Science* **340**, 1560–1563 (2013).
49. J. Austermann, J. X. Mitrovica, P. Huybers, A. Rovere, Detection of a dynamic topography signal in last interglacial sea-level records. *Sci. Adv.* **3**, e1700457 (2017).
50. A. Hollyday, J. Austermann, A. Lloyd, M. Hoggard, F. Richards, A. Rovere, A revised estimate of early Pliocene global mean sea level using geodynamic models of the Patagonian slab window. *Geochem. Geophys. Geosyst.* **24**, e2022GC010648 (2023).
51. L. E. Lisiecki, M. E. Raymo, A Pliocene-Pleistocene stack of 57 globally distributed benthic $\delta^{18}$O records. *Paleoceanography* **20**, 10.1029/2004PA001071. (2005).
52. B. de Boer, A. M. Haywood, A. M. Dolan, S. J. Hunter, C. L. Prescott, The transient response of ice volume to orbital forcing during the warm late Pliocene. *Geophys. Res. Lett.* **44**, 10486–10494 (2017).
53. B. Dyer, J. Austermann, W. J. D'Andrea, R. C. Creel, M. R. Sandstrom, M. Cashman, A. Rovere, M. E. Raymo, Sea-level trends across The Bahamas constrain peak last interglacial ice melt. *Proc. Natl. Acad. Sci.* **118** (2021).
54. G. Meehl, T. Stocke, W. Collins, P. Friedlingstein, A. Gaye, J. Gregory, A. Kitoh, R. Knutti, J. Murphy, A. Noda, S. Raper, I. Watterson, A. Weaver, Z.-C. Zhao, 2007: Global Climate Projections, in *Climate Change 2007: The Physical Science Basis. Contribution of Working Group I to the Fourth Assessment Report of the Intergovernmental Panel on Climate Change*, *S. Solomon*, D. Qin, M. Manning, Z. Chen, M. Marquis, K. Averyt, M. Tignor, H. Miller, Eds. (Cambridge Univ. Press, 2007).
55. T. Blackburn, G. Edwards, S. Tulaczyk, M. Scudder, G. Piccione, B. Hallet, N. McLean, J. Zachos, B. Cheney, J. Babbe, Ice retreat in Wilkes Basin of East Antarctica during a warm interglacial. *Nature* **583**, 554–559 (2020).
56. A. M. Dolan, B. De Boer, J. Bernales, D. J. Hill, A. M. Haywood, High climate model dependency of Pliocene Antarctic ice-sheet predictions. *Nat. Commun.* **9**, 2799 (2018).
57. T. L. Edwards, S. Nowicki, B. Marzeion, R. Hock, H. Goelzer, H. Seroussi, N. C. Jourdain, D. A. Slater, F. E. Turner, C. J. Smith, C. M. McKenna, E. Simon, A. Abe-Ouchi, J. M. Gregory, E. Larour, W. H. Lipscomb, A. J. Payne, A. Shepherd, C. Agosta, P. Alexander, T. Albrecht, B. Anderson, X. Asay-Davis, A. Aschwanden, A. Barthel, A. Bliss, R. Calov, C. Chambers, N.

Champollion, Y. Choi, R. Cullather, J. Cuzzone, C. Dumas, D. Felikson, X. Fettweis, K. Fujita, B. K. Galton-Fenzi, R. Gladstone, N. R. Golledge, R. Greve, T. Hattermann, M. J. Hoffman, A. Humbert, M. Huss, P. Huybrechts, W. Immerzeel, T. Kleiner, P. Kraaijenbrink, S. Le Clec'h, V. Lee, G. R. Leguy, C. M. Little, D. P. Lowry, J.-H. Malles, D. F. Martin, F. Maussion, M. Morlighem, J. F. O'Neill, I. Nias, F. Pattyn, T. Pelle, S. F. Price, A. Quiquet, V. Radić, R. Reese, D. R. Rounce, M. Rückamp, A. Sakai, C. Shafer, N.-J. Schlegel, S. Shannon, R. S. Smith, F. Straneo, S. Sun, L. Tarasov, L. D. Trusel, J. Van Breedam, R. S. W. van de Wal, M. van den Broeke, R. Winkelmann, H. Zekollari, C. Zhao, T. Zhang, T. Zwinger, Projected land ice contributions to twenty-first-century sea level rise. *Nature* **593**, 74–82 (2021).

58. B. de Boer, A. M. Dolan, J. Bernales, E. Gasson, H. Goelzer, N. R. Golledge, J. Sutter, P. Huybrechts, G. Lohmann, I. Rogozhina, A. Abe-Ouchi, F. Saito, and R. S. W. van de Wal, Simulating the antarctic ice sheet in the late-Pliocene warm period: PLISMIP-ANT, an ice-sheet model intercomparison project. *Cryosphere* **9**, 881–903 (2015).
59. M. Oppenheimer, B. Glavovic, J. Hinkel, R. van de Wal, A. Magnan, A. Abd-Elgawad, R. Cai, M. Cifuentes-Jara, R. DeConto, T. Ghosh, J. Hay, F. Isla, B. Marzeion, B. Meyssignac, Z. Sebesvari, Sea level rise and implications for low-lying islands, coasts and communities, in *IPCC Special Report on the Ocean and Cryosphere in a Changing Climate*, H.-O. Pörtner, D. Roberts, V. Masson-Delmotte, P. Zhai, M. Tignor, E. Poloczanska, K. Mintenbeck, A. Alegriìa, M. Nicolai, A. Okem, J. Petzold, B. Rama, N. Weyer, Eds. (Cambridge Univ. Press, 2019), pp. 321–445.
60. M. A. Kominz, J. Browning, K. Miller, P. Sugarman, S. Mizintseva, C. Scotese, Late Cretaceous to Miocene sea-level estimates from the New Jersey and Delaware coastal plain coreholes: An error analysis. *Basin Res.* **20**, 211–226 (2008).
61. F. D. Richards, M. J. Hoggard, N. White, S. Ghelichkhan, Quantifying the relationship between short-wavelength dynamic topography and thermomechanical structure of the upper mantle using calibrated parameterization of *Anelasticity J. Geophys. Res. Solid Earth* **125**, e2019JB019062 (2020).
62. A. Schaeffer, S. Lebedev, Global shear speed structure of the upper mantle and transition zone. *Geophys. J. Int.* **194**, 417–449 (2013).
63. A. Schaeffer, S. Lebedev, Imaging the North American continent using waveform inversion of global and USArray data. *Earth Planet. Sci. Lett.* **402**, 26–41 (2014).

64. N. L. Celli, S. Lebedev, A. J. Schaeffer, C. Gaina, African cratonic lithosphere carved by mantle plumes. *Nat. Commun.* **11**, 92 (2020).
65. N. L. Celli, S. Lebedev, A. J. Schaeffer, M. Ravenna, C. Gaina, The upper mantle beneath the South Atlantic Ocean, South America and Africa from waveform tomography with massive data sets. *Geophys. J. Int.* **221**, 178–204 (2020).
66. M. J. Hoggard, K. Czarnota, F. D. Richards, D. L. Huston, A. L. Jaques, S. Ghelichkhan, Global distribution of sediment-hosted metals controlled by craton edge stability. *Nat. Geosci.* **13**, 504–510 (2020).
67. J. Austermann, M. J. Hoggard, K. Latychev, F. D. Richards, J. X. Mitrovica, The effect of lateral variations in Earth structure on Last Interglacial sea level. *Geophys. J. Int.* **227**, 1938–1960 (2021).
68. L. Stixrude, C. Lithgow-Bertelloni, Thermodynamics of mantle minerals—II: Phase equilibria. *Geophys. J. Int.* **184**, 1180–1213 (2011).
69. B. S. A. Schuberth, H. P. Bunge, Tomographic filtering of high-resolution mantle circulation models: Can seismic heterogeneity be explained by temperature alone? *Geochem. Geophys. Geosyst.* **10**, Q05W03 (2009).
70. J. Matas, M. S. Bukowinski, On the anelastic contribution to the temperature dependence of lower mantle seismic velocities. *Earth Planet. Sci. Lett.* **259**, 51–65 (2007).
71. D. R. Davies, S. Ghelichkhan, M. Hoggard, A. Valentine, F. D. Richards, Observations and models of dynamic topography: Current status and future directions. *EarthArXiv* 10.31223/X55W5T. (2022).
72. T. H. Jordan, Composition and development of the continental tectosphere. *Nature* **274**, 544–548 (1978).
73. S. S. Shapiro, B. H. Hager, T. H. Jordan, The continental tectosphere and Earth's long-wavelength gravity field. *Lithos* **48**, 135–152 (1999).
74. H. Fei, U. Faul, T. Katsura, The grain growth kinetics of bridgmanite at the topmost lower mantle. *Earth Planet. Sci. Lett.* **561**, 116820 (2021).
75. R. Moucha, A. M. Forte, J. X. Mitrovica, D. B. Rowley, S. Quéré, N. A. Simmons, S. P. Grand, Dynamic topography and long-term sea-level variations: There is no such thing as a stable continental platform. *Earth Planet. Sci. Lett.* **271**, 101–108 (2008).
76. P. Glišović, A. M. Forte, Importance of initial buoyancy field on evolution of mantle thermal structure: Implications of surface boundary conditions. *Geosci. Front.* **6**, 3–22 (2015).

77. C. DeMets, R. G. Gordon, D. F. Argus, Geologically current plate motions. *Geophys. J. Int.* **181**, 1–80 (2010).
78. M. Seton, R. D. Müller, S. Zahirovic, C. Gaina, T. Torsvik, G. Shephard, A. Talsma, M. Gurnis, M. Turner, S. Maus, M. Chandler, Global continental and ocean basin reconstructions since 200Ma. *Earth Sci. Rev.* **113**, 212–270 (2012).
79. R. A. Kendall, J. X. Mitrovica, G. A. Milne, On post-glacial sea level—II: Numerical formulation and comparative results on spherically symmetric models. *Geophys. J. Int.* **161**, 679–706 (2005).
80. W. R. Peltier, Global glacial isostasy and the surface of the ice-age Earth: The ICE-5G (VM2) model and GRACE. *Annu. Rev. Earth Planet. Sci.* **32**, 111–149 (2004).
81. J. X. Mitrovica, J. Wahr, I. Matsuyama, A. Paulson, The rotational stability of an ice-age Earth. *Geophys. J. Int.* **161**, 491–506 (2005).
82. D. P. Kingma, J. Ba, Adam: A method for stochastic optimization. arXiv:1412.6980 [quant-ph] (2014).
83. J. Salvatier, T. V. Wiecki, C. Fonnesbeck, Probabilistic programming in Python using PyMC3. *PeerJ Computer Science* **2**, e55 (2016).
84. A. Shepherd, E. Ivins, E. Rignot, B. Smith, M. Van Den Broeke, I. Velicogna, P. Whitehouse, K. Briggs, I. Joughin, G. Krinner, S. Nowicki, T. Payne, T. Scambos, N. Schlegel, A. Geruo, C. Agosta, A. Ahlstrøm, G. Babonis, V. Barletta, A. Blazquez, J. Bonin, B. Csatho, R. Cullather, D. Felikson, X. Fettweis, R. Forsberg, H. Gallee, A. Gardner, L. Gilbert, A. Groh, B. Gunter, E. Hanna, C. Harig, V. Helm, A. Horvath, M. Horwath, S. Khan, K. K. Kjeldsen, H. Konrad, P. Langen, B. Lecavalier, B. Loomis, S. Luthcke, M. McMillan, D. Melini, S. Mernild, Y. Mohajerani, P. Moore, J. Mouginot, G. Moyano, A. Muir, T. Nagler, G. Nield, J. Nilsson, B. Noel, I. Otosaka, M. E. Pattle, W. R. Peltier, N. Pie, R. Rietbroek, H. Rott, L. Sandberg-Sørensen, I. Sasgen, H. Save, B. Scheuchl, E. Schrama, L. Schröder, K.-W. Seo, S. Simonsen, T. Slater, G. Spada, T. Sutterley, M. Talpe, L. Tarasov, W. J. van de Berg, W. van der Wal, M. van Wessem, B. D. Vishwakarma, D. Wiese, B. Wouters, Mass balance of the Antarctic Ice Sheet from 1992 to 2017. *Nature* **558**, 219–222 (2018).
85. W. Bangerth, J. Dannberg, R. Gassmöller, T. Heister, Aspect v2.1.0 (2019).
86. M. J. Hoggard, J. Winterbourne, K. Czarnota, N. White, Oceanic residual depth measurements, the plate cooling model, and global dynamic topography. *J. Geophys. Res. Solid Earth* **122**, 2328–2372 (2017).

87. H. J. Dowsett, T. M. Cronin, High eustatic sea level during the middle Pliocene:Evidence from the southeastern U.S. Atlantic Coastal Plain. *Geology* **18**, 435–438 (1990).
88. K. G. Miller, J. D. Wright, J. V. Browning, A. Kulpecz, M. Kominz, T. R. Naish, B. S. Cramer, Y. Rosenthal, W. R. Peltier, S. Sosdian, High tide of the warm Pliocene: Implications of global sea level for Antarctic deglaciation. *Geology* **40**, 407–410 (2012).
89. R. K. Workman, S. R. Hart, Major and trace element composition of the depleted MORB mantle (DMM). *Earth Planet. Sci. Lett.* **231**, 53–72 (2005).
90. I. Tolstikhin, A. W. Hofmann, Early crust on top of the Earth's core. *Phys. Earth Planet. Int.* **148**, 109–130 (2005).
91. S. Labrosse, J. Hernlund, N. Coltice, A crystallizing dense magma ocean at the base of the Earth's mantle. *Nature* **450**, 866–869 (2007).
92. C.-T. A. Lee, P. Luffi, T. Höink, J. Li, R. Dasgupta, J. Hernlund, Upside-down differentiation and generation of a 'primordial' lower mantle. *Nature* **463**, 930–933 (2010).
93. A. M. Dziewonski, D. L. Anderson, Preliminary reference Earth model. *Phys. Earth Planet. Int.* **25**, 297–356 (1981).
94. V. Corrieu, C. Thoraval, Y. Ricard, Mantle dynamics and geoid Green functions. *Geophys. J. Int.* **120**, 516–523 (1995).
95. P. J. Tackley, Dynamics and evolution of the deep mantle resulting from thermal, chemical, phase and melting effects. *Earth-Sci. Rev.* **110**, 1–25 (2012).
96. E. Mulyukova, B. Steinberger, M. Dabrowski, S. V. Sobolev, Survival of LLSVPs for billions of years in a vigorously convecting mantle: Replenishment and destruction of chemical anomaly. *J. Geophys. Res. Solid Earth* **120**, 3824–3847 (2015).
97. T. D. Jones, R. R. Maguire, P. E. van Keken, J. Ritsema, P. Koelemeijer, Subducted oceanic crust as the origin of seismically slow lower-mantle structures. *Prog. Earth Planet. Sci.* **7**, 1–16 (2020).
98. B. Steinberger, A. R. Calderwood, Models of large-scale viscous flow in the Earth's mantle with constraints from mineral physics and surface observations. *Geophys. J. Int.* **167**, 1461–1481 (2006).
